# Cultivar-Dependent Effects of Basil–Common Bean Intercropping on Mineral and Lipophilic Antioxidant Composition

**DOI:** 10.3390/plants15213443

**Published:** 2026-11-05

**Authors:** Klaudija Carović-Stanko, Boris Lazarević, Tomislav Javornik, Monika Vidak, Kristina Kljak

**Affiliations:** 1 Centre of Excellence for Biodiversity and Molecular Plant Breeding (CoE CroP-BioDiv), HR-10000 Zagreb, Croatia; blazarevic@agr.hr (B.L.); tjavornik@agr.hr (T.J.); vidak@net.hr (M.V.); kkljak@agr.hr (K.K.); 2 Department of Plant Biodiversity, Faculty of Agriculture, University of Zagreb, Svetošimunska Cesta 25, HR-10000 Zagreb, Croatia; 3 Department of Plant Nutrition, Faculty of Agriculture, University of Zagreb, Svetošimunska Cesta 25, HR-10000 Zagreb, Croatia; 4 Department of Animal Nutrition, Faculty of Agriculture, University of Zagreb, Svetošimunska Cesta 25, HR-10000 Zagreb, Croatia

**Keywords:** *Ocimum basilicum* L., *Phaseolus vulgaris* L., carotenoids, tocopherols, cultivar-dependent response

## Abstract

Intercropping can affect nutrient availability and plant biochemical composition, but information on these effects in medicinal and aromatic plants is still limited. This study evaluated the effects of intercropping with common bean (*Phaseolus vulgaris* L.) on mineral composition, carotenoids, and tocopherols in two basil (*Ocimum basilicum* L.) cultivars, ‘Genovese’ and ‘Sweet basil’, grown at different basil–bean ratios. Intercropping significantly affected all analyzed minerals, although the response differed between cultivars for several elements. ‘Genovese’ showed increases in Zn, Mn, and Cu, while Zn content in ‘Sweet basil’ remained relatively stable. In contrast, N, P, and Fe concentrations decreased in some treatments. Total carotenoids and total lutein decreased under intercropping in both cultivars, whereas β-carotene responses were less consistent and depended on cultivar and intercropping ratio. In contrast, total tocopherols and α-tocopherol generally increased under intercropping. Zn, Mn, and Cu were negatively correlated with several carotenoids but positively correlated with tocopherols. PCA separated monocropped from most intercropped samples and showed that both mineral and antioxidant variables contributed to sample differentiation. These findings show that the compositional response of basil to common bean intercropping depends on cultivar, intercropping ratio, and the group of compounds considered.

## 1. Introduction

Intercropping is a long-established agricultural practice in which two or more crop species are grown together for a substantial part of their growing period. By increasing crop diversity, it can improve resource use, nutrient cycling, and overall agroecosystem functioning while reducing dependence on external inputs [1,2]. Although intercropping research has traditionally focused on yield and land-use efficiency, it has recently received increasing attention for its effects on crop nutritional quality and bioactive compounds [3]. However, the links between changes in plant mineral and antioxidant composition under intercropping remain insufficiently explored.

Legumes are widely used in intercropping systems because of their capacity for symbiotic N_2_ fixation and their influence on nutrient availability to associated crops. Common bean (*Phaseolus vulgaris* L.) is among the legume species frequently used in intercrops [4]. In addition to biological N_2_ fixation, common bean can modify rhizosphere processes and nutrient acquisition by associated crops [5]. These interactions are not exclusively facilitative, as competition for below- and aboveground resources can occur simultaneously and influence the physiological responses of plants within the intercropping system [6].

Basil (*Ocimum basilicum* L.) is a widely cultivated plant used for culinary and medicinal purposes. Its leaves are rich in bioactive compounds, including phenolic acids, flavonoids, chlorophylls, carotenoids, tocopherols, and essential-oil constituents, which contribute to both its nutritional quality and antioxidant potential [7,8,9]. The accumulation of these compounds depends strongly on genotype and is also influenced by cultivation practices and environmental conditions [7,10,11]. Among these compounds, carotenoids and tocopherols are important components of the lipophilic antioxidant system. Carotenoids are involved in light harvesting and photoprotection, whereas tocopherols protect membrane lipids from oxidative damage [8].

Intercropping modifies canopy architecture, light interception, belowground competition, and nutrient dynamics and may therefore affect the biosynthesis and accumulation of lipophilic antioxidants. Previous studies have demonstrated that intercropping can modify plant growth and the accumulation of bioactive compounds, including carotenoids. However, the direction and magnitude of these responses depend on crop combination, spatial arrangement, planting density, and environmental conditions [2,12,13]. In basil–common bean intercrops, different cropping patterns have also been shown to affect mineral nutrient uptake and the quantity and composition of basil essential oil [9], suggesting that the biochemical composition of basil may respond to interactions between the two component crops. However, little is known about the effects of basil–common bean intercropping on carotenoid and tocopherol accumulation or whether these responses differ between basil cultivars.

Mineral composition is an important component of basil quality and represents another potential response to intercropping. Essential macro- and micronutrients contribute to plant growth and metabolism, while the mineral profile of edible leaves is also relevant from a nutritional perspective. Intercropping can modify nutrient acquisition and mineral content in plant tissues through changes in root interactions, nutrient availability, and competition or facilitation between component crops [13,14]. In peanut–maize intercropping, for example, Fe, Zn, and Mn concentrations increased in peanut shoots [15], while cereal–legume intercropping has been shown to affect the acquisition, translocation, and allocation of mineral nutrients, particularly P, Fe, and Zn [14]. Importantly, changes in nutrient acquisition are not necessarily reflected by corresponding changes in tissue mineral content, because the final concentration also depends on biomass production, nutrient translocation, and allocation within the plant [14]. In basil–common bean systems, Rezaei-Chiyaneh et al. [9] reported changes in the uptake of several macro- and micronutrients depending on planting pattern and management. However, nutrient uptake and mineral content in basil leaves represent different aspects of plant mineral nutrition, and information on cultivar-dependent changes in leaf mineral content under common bean intercropping remains limited.

Mineral nutrition may also be associated with the antioxidant response to intercropping. Several mineral elements are involved in photosynthetic processes and cellular redox regulation, acting as structural components, enzyme cofactors, or participants in photosynthetic electron transport [16,17]. Changes in mineral status may therefore be accompanied by changes in chloroplast antioxidants such as carotenoids and tocopherols [18]. However, the relationship between changes in mineral composition and lipophilic antioxidants under intercropping has received little attention, particularly in medicinal and aromatic plants.

Therefore, the objective of this study was to investigate the effects of intercropping with common bean (*P. vulgaris* L., ‘Biser’, market class Navy bean) on the mineral composition and carotenoid and tocopherol contents in two basil cultivars (‘Genovese’ and ‘Sweet basil’). We hypothesized that (i) intercropping would alter the mineral composition and carotenoid and tocopherol contents of basil, (ii) the magnitude of these responses would depend on the proportion of common bean in the intercrop, and (iii) the response to intercropping would differ between basil cultivars.

## 2. Results

### 2.1. Mineral Composition of Basil Leaves

The mixed-model analysis revealed that the intercropping ratio significantly affected the contents of all analyzed minerals (N, P, K, Ca, Mg, Fe, Zn, Mn, and Cu) (Table 1). Intercropping ratio effects were highly significant for K, Ca, Mg, Zn, Mn, and Cu (*p* < 0.0001), while significant effects were also observed for N (*p* = 0.0058), P (*p* = 0.0090), and Fe (*p* = 0.0032). Basil cultivars significantly differed in most mineral elements, particularly Ca, Mn, Zn, Cu, and Mg, whereas no significant cultivar effect was observed for P and K. Significant cultivar effects were also detected for N (*p* = 0.0009) and Fe (*p* = 0.0214). A significant cultivar × intercropping ratio interaction was detected for K, Ca, Mg, Zn, Mn, and Cu, showing that the two basil cultivars responded differently to the various intercropping ratios. The interaction was highly significant for Mg, Cu, Mn and Zn (*p* < 0.0001), and was also significant for Ca (*p* = 0.0008), and K (*p* = 0.0077) (Table 1), indicating cultivar-specific differences in mineral contents under intercropping conditions. No significant cultivar × intercropping ratio interaction was detected for N, P, or Fe.

Nitrogen content was significantly affected by cultivar and intercropping ratio. ‘Sweet basil’ generally exhibited higher N contents than ‘Genovese’ (38.32 and 36.64 g kg^−1^ DM, respectively; Table 1). When averaged across cultivars, N content was highest in the control (38.67 g kg^−1^ DM), decreased significantly at the 75:25 and 50:50 ratios (36.71 and 36.56 g kg^−1^ DM, respectively), and was intermediate at the 25:75 ratio (38.00 g kg^−1^ DM). Intercropping affected phosphorus content, but neither the cultivar effect nor the cultivar × intercropping interaction was significant. The control had the highest mean P content (3.79 g kg^−1^ DM), while the 50:50 and 25:75 ratios had significantly lower values (3.50 and 3.55 g kg^−1^ DM, respectively); the 75:25 ratio was intermediate (3.66 g kg^−1^ DM) (Table 1).

**Figure 1:**
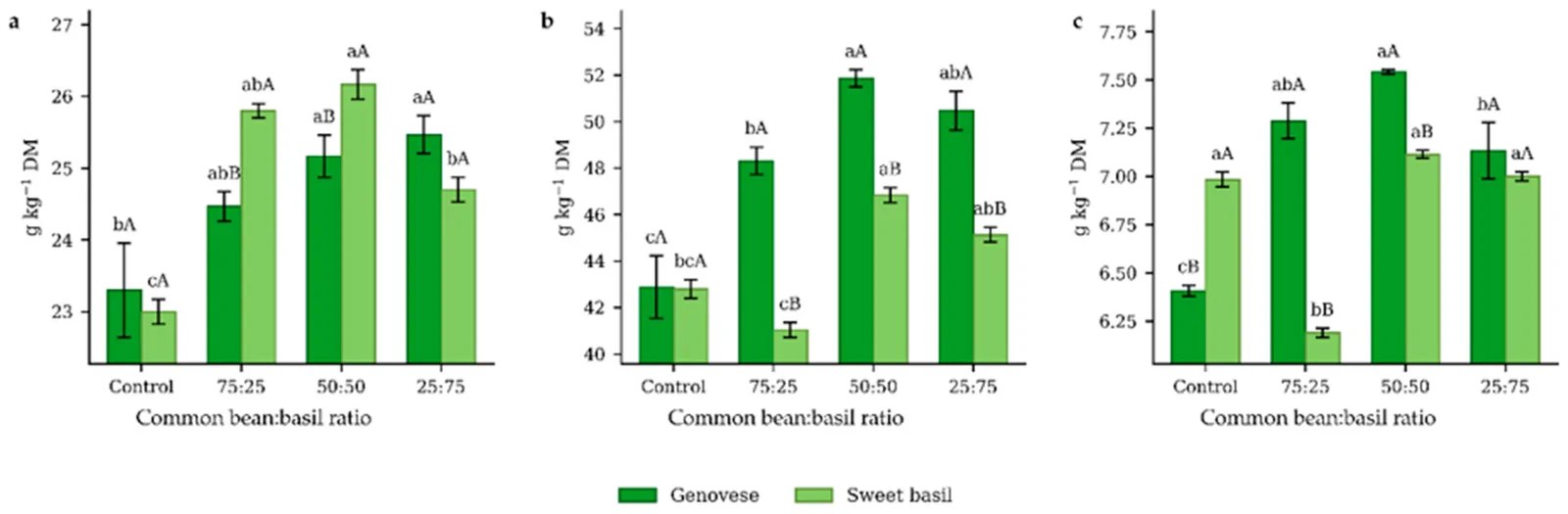
Significant cultivar × intercropping ratio interactions for macroelements: (**a**) K, (**b**) Ca, and (**c**) Mg in basil leaves. Bars represent LSMeans ± SE (model-based standard error of the least-squares mean calculated from individual plot observations; *n* = 3). Lowercase letters indicate significant differences among intercropping ratios within a cultivar, and uppercase letters indicate significant differences between cultivars within the same intercropping ratio, based on Tukey-adjusted comparisons of least-squares means (*p* < 0.05).

Potassium content showed a significant cultivar × intercropping ratio interaction (Table 1, Figure 1). K content was generally higher under intercropping than in monoculture, although responses differed between cultivars and intercropping ratios. In ‘Genovese’, K content increased from 23.30 g kg^−1^ DM in the control to 25.17 and 25.47 g kg^−1^ DM at the 50:50 and 25:75 ratios, respectively. The 75:25 ratio showed an intermediate value of 24.47 g kg^−1^ DM. In ‘Sweet basil’, K increased from 23.00 g kg^−1^ DM in the control to a maximum of 26.17 g kg^−1^ DM at the 50:50 ratio. Values of 25.80, 26.17, and 24.70 g kg^−1^ DM were recorded at the 75:25, 50:50, and 25:75 ratios, respectively; all three intercropping treatments were significantly higher than the control. The 50:50 ratio also resulted in higher K content than the 25:75 ratio. ‘Sweet basil’ had significantly higher K content than ‘Genovese’ at the 75:25 and 50:50 ratios, whereas the cultivars did not differ significantly in the control or at the 25:75 ratio.

Calcium content showed a significant cultivar × intercropping ratio interaction (Table 1, Figure 1). Although the two cultivars had similar Ca contents under monoculture, they responded differently to intercropping with common bean. In ‘Genovese’, Ca increased from 42.88 g kg^−1^ DM in monoculture to 48.30, 51.86, and 50.47 g kg^−1^ DM at the 75:25, 50:50, and 25:75 ratios, respectively. All three intercropping ratios resulted in significantly higher Ca contents than the control; the 50:50 ratio was also significantly higher than 75:25, whereas 25:75 did not differ from either intercropping treatment. In contrast, ‘Sweet basil’ showed no significant change in Ca content at the 75:25 ratio compared with the control (41.05 vs. 42.80 g kg^−1^ DM). Ca content increased to 46.84 g kg^−1^ DM at the 50:50 ratio, which was significantly higher than the control and 75:25 treatment, whereas the value at 25:75 (45.13 g kg^−1^ DM) was intermediate. The cultivars did not differ in Ca content under monoculture, whereas ‘Genovese’ had significantly higher Ca contents than ‘Sweet basil’ at all three intercropping ratios.

Mg content also showed a significant cultivar × intercropping ratio interaction (Table 1, Figure 1). In ‘Genovese’, Mg increased from 6.41 g kg^−1^ DM in monoculture to 7.29 g kg^−1^ DM at the 75:25 ratio and reached a maximum of 7.54 g kg^−1^ DM at the 50:50 ratio. All intercropping treatments had significantly higher Mg contents than the control, while the 50:50 ratio was also significantly higher than 25:75. In contrast, ‘Sweet basil’ showed a different response: Mg decreased from 6.98 g kg^−1^ DM in monoculture to 6.19 g kg^−1^ DM at the 75:25 ratio, whereas the 50:50 and 25:75 ratios resulted in values of 7.11 and 7.00 g kg^−1^ DM, respectively. Only the 75:25 treatment differed significantly from the control and the other intercropping ratios. ‘Sweet basil’ had higher Mg content than ‘Genovese’ in monoculture, whereas ‘Genovese’ had higher contents at the 75:25 and 50:50 ratios.

Iron content was significantly influenced by cultivar and intercropping ratio, without a significant cultivar × intercropping ratio interaction (Table 1). Overall, ‘Sweet basil’ contained more Fe than ‘Genovese’ (1734.07 vs. 1617.53 mg kg^−1^ DM). Intercropping generally reduced Fe content, with the control showing the highest mean content (1837.90 mg kg^−1^ DM). Fe contents at the 75:25 and 50:50 ratios (1544.85 and 1634.00 mg kg^−1^ DM, respectively) were significantly lower than the control, whereas the 25:75 ratio (1686.44 mg kg^−1^ DM) was intermediate and did not differ significantly from either group.

**Figure 2:**
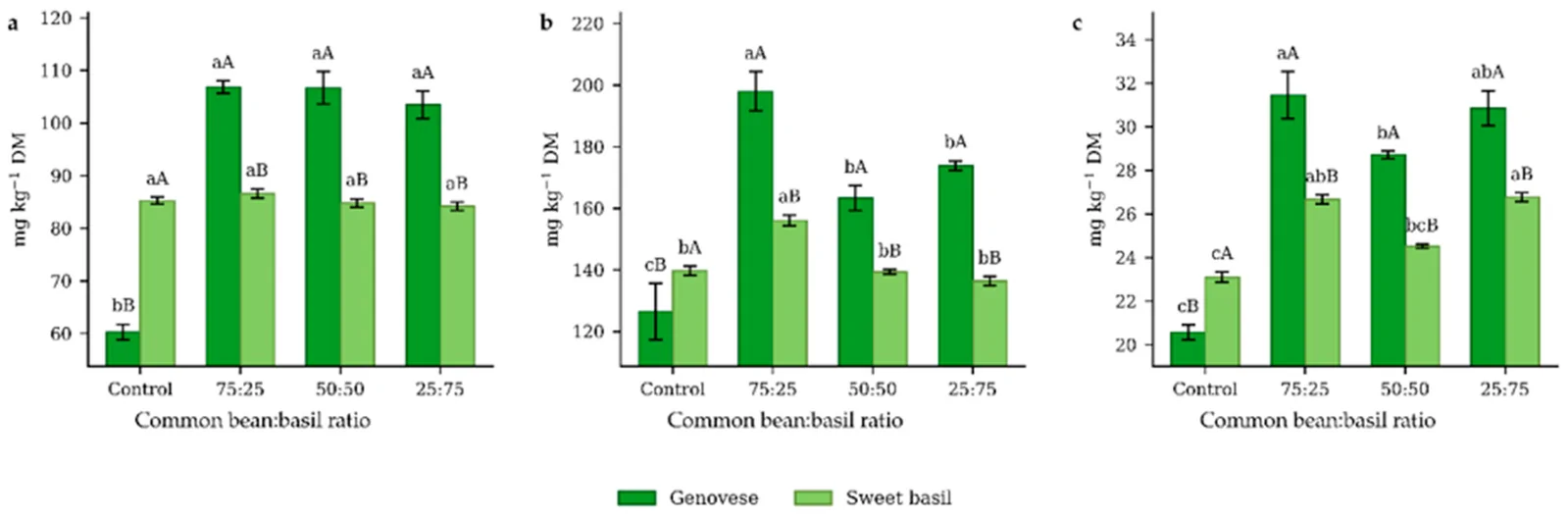
Significant cultivar × intercropping ratio interactions for microelements: (**a**) Zn, (**b**) Mn, and (**c**) Cu in basil leaves. Bars represent LSMeans ± SE (model-based standard error of the least-squares mean calculated from individual plot observations; *n* = 3). Lowercase letters indicate significant differences among intercropping ratios within a cultivar, and uppercase letters indicate significant differences between cultivars within the same intercropping ratio, based on Tukey-adjusted comparisons of least-squares means (*p* < 0.05).

Among the analyzed micronutrients, Zn showed a pronounced cultivar-specific response, with significant effects of cultivar, intercropping ratio, and their interaction (all *p* < 0.0001; Table 1, Figure 2). ‘Genovese’ contained only 60.30 mg kg^−1^ DM Zn in monoculture, but intercropping increased Zn content to 106.84, 106.68, and 103.47 mg kg^−1^ DM at the 75:25, 50:50, and 25:75 ratios, respectively, corresponding to increases of approximately 72–77% compared with the control. All three intercropping ratios resulted in significantly higher Zn contents than the ‘Genovese’ control but did not differ from one another. In contrast, Zn content in ‘Sweet basil’ remained relatively stable across treatments, ranging from 84.18 to 86.58 mg kg^−1^ DM compared with 85.23 mg kg^−1^ DM in the control, with no significant differences among intercropping ratios. ‘Sweet basil’ had significantly higher Zn content than ‘Genovese’ in monoculture, whereas ‘Genovese’ had significantly higher Zn contents at all three intercropping ratios.

Similarly, Mn and Cu contents were significantly affected by cultivar, intercropping ratio, and their interaction (all *p* < 0.0001; Table 1, Figure 2). In ‘Genovese’, Mn increased markedly from 126.49 mg kg^−1^ DM in monoculture to a maximum of 197.97 mg kg^−1^ DM at the 75:25 ratio. The 50:50 and 25:75 ratios also increased Mn content to 163.36 and 173.90 mg kg^−1^ DM, respectively. All three intercropping ratios had significantly higher Mn contents than the control, with the 75:25 ratio also significantly higher than the 50:50 and 25:75 ratios. In contrast, ‘Sweet basil’ showed a significant increase only at the 75:25 ratio, from 139.73 mg kg^−1^ DM in the control to 156.11 mg kg^−1^ DM at 75:25, while values at 50:50 and 25:75 (139.37 and 136.45 mg kg^−1^ DM, respectively) did not differ significantly from the control. ‘Sweet basil’ had a higher Mn content than ‘Genovese’ in monoculture, whereas ‘Genovese’ had higher Mn contents at all three intercropping ratios.

A comparable cultivar-dependent pattern was observed for Cu. In ‘Genovese’, Cu increased from 20.57 mg kg^−1^ DM in the control to 31.46, 28.72, and 30.85 mg kg^−1^ DM at the 75:25, 50:50, and 25:75 ratios, respectively. All three intercropping ratios resulted in significantly higher Cu contents than the control, while the 75:25 ratio was also significantly higher than the 50:50 ratio. ‘Sweet basil’ showed a different response, from 23.10 mg kg^−1^ DM in the control to 26.66 and 26.76 mg kg^−1^ DM at the 75:25 and 25:75 ratios, respectively, both of which were significantly higher than the control. The 50:50 ratio resulted in 24.53 mg kg^−1^ DM and did not differ significantly from the control. As observed for Mn, ‘Sweet basil’ had higher Cu content than ‘Genovese’ in monoculture, whereas ‘Genovese’ had higher Cu contents at all three intercropping ratios.

### 2.2. Carotenoid and Tocopherol Composition

**Figure 3:**
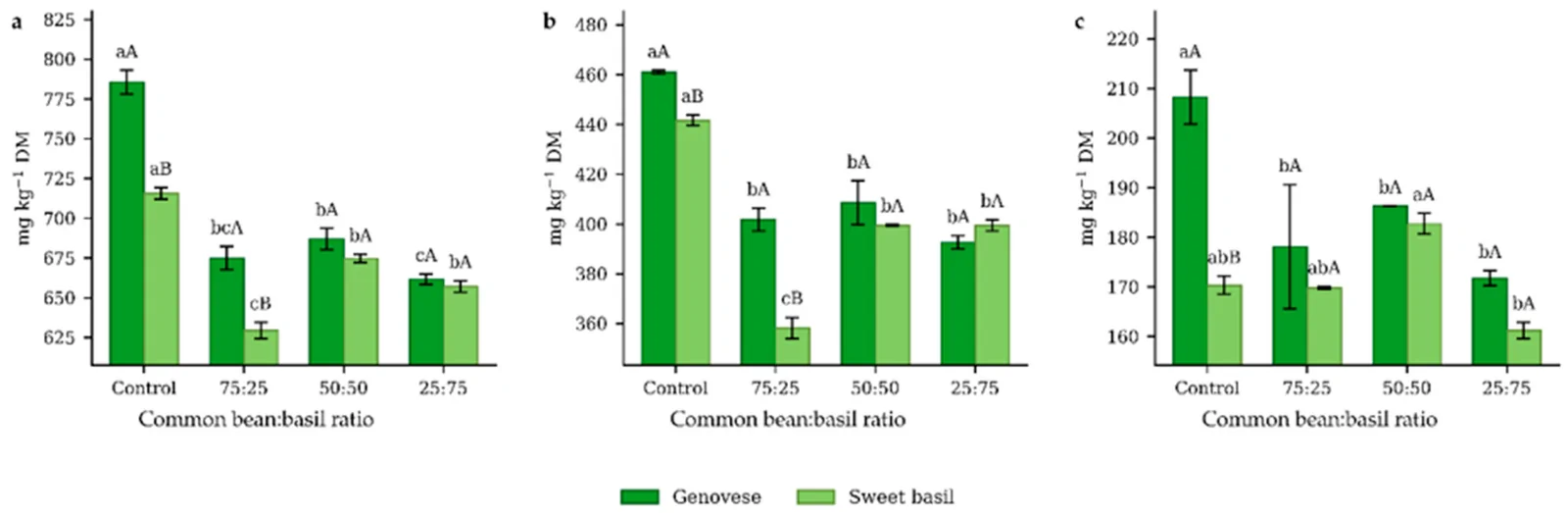
Significant cultivar × intercropping ratio interactions for major carotenoids: (**a**) total carotenoids, (**b**) total lutein, and (**c**) total β-carotene in basil leaves. Bars represent LSMeans ± SE (model-based standard error of the least-squares mean calculated from individual plot observations; *n* = 3). Lowercase letters indicate significant differences among intercropping ratios within a cultivar, and uppercase letters indicate significant differences between cultivars within the same intercropping ratio, based on Tukey-adjusted comparisons of least-squares means (*p* < 0.05).

Carotenoid content was affected by both basil cultivar and intercropping ratio. Significant cultivar × intercropping ratio interactions were detected for total carotenoids (*p* < 0.0001), total lutein (*p* = 0.0004), and total β-carotene (*p* = 0.0094) (Table 2, Figure 3), indicating that the two cultivars differed in their response to intercropping. Both cultivar and intercropping ratio also had significant main effects on all three total carotenoid variables. Total carotenoid content was highest in monocropped ‘Genovese’ (785.66 mg kg^−1^ DM), followed by monocropped ‘Sweet basil’ (715.58 mg kg^−1^ DM; Table 2). Intercropping reduced total carotenoid contents in both cultivars, although the magnitude of this response depended on cultivar and bean ratio. In ‘Genovese’, all three intercropping ratios significantly reduced total carotenoid content compared with monoculture, from 785.66 mg kg^−1^ DM to 674.87, 686.96, and 661.54 mg kg^−1^ DM at the 75:25, 50:50, and 25:75 ratios, respectively. In ‘Sweet basil’, total carotenoid content also decreased significantly under all three intercropping treatments, from 715.58 mg kg^−1^ DM in monoculture to 629.48, 674.74, and 657.12 mg kg^−1^ DM at the 75:25, 50:50, and 25:75 ratios, respectively. ‘Genovese’ had significantly higher total carotenoid content than ‘Sweet basil’ in monoculture and at the 75:25 ratio, whereas the cultivars did not differ significantly at the 50:50 or 25:75 ratios.

A similar response was observed for total lutein, with cultivar, intercropping, and their interaction all highly significant (*p* = 0.0001, *p* < 0.0001, and *p* = 0.0004, respectively). Monocropped ‘Genovese’ and ‘Sweet basil’ contained 460.99 and 441.57 mg kg^−1^ DM total lutein, respectively, whereas the lowest content was observed in ‘Sweet basil’ grown at 75:25 ratio (358.24 mg kg^−1^ DM; Table 2, Figure 3). Intercropping significantly reduced total lutein in both cultivars. In ‘Genovese’, values ranged from 392.58 to 408.51 mg kg^−1^ DM under intercropping and all three intercropping ratios had significantly lower total lutein contents than the control, with no significant differences among the intercropping treatments. In ‘Sweet basil’, total lutein decreased to 358.24, 399.45, and 399.42 mg kg^−1^ DM at the 75:25, 50:50, and 25:75 ratios, respectively. All three values were significantly lower than the control, while the 75:25 treatment was also significantly lower than the 50:50 and 25:75 treatments. ‘Genovese’ had significantly higher total lutein content than ‘Sweet basil’ in monoculture and at the 75:25 ratio, whereas the cultivars did not differ at the 50:50 or 25:75 ratios. Trans-lutein was the predominant lutein fraction and followed a similar response to intercropping, with a significant cultivar × intercropping ratio interaction (*p* = 0.0007; Table 2).

Total β-carotene was significantly affected by cultivar (*p* = 0.0004), intercropping ratio (*p* = 0.0009), and their interaction (*p* = 0.0094) (Table 2, Figure 3). ‘Genovese’ grown in monoculture showed the highest total β-carotene content (208.28 mg kg^−1^ DM). However, the response to intercropping differed between cultivars. In ‘Genovese’, total β-carotene decreased to 178.09, 186.30, and 171.74 mg kg^−1^ DM at the 75:25, 50:50, and 25:75 ratios, respectively. All three intercropping treatments had significantly lower total β-carotene contents than the control, but did not differ significantly from one another. In ‘Sweet basil’, the response was less consistent: total β-carotene was 170.34 mg kg^−1^ DM in monoculture, remained similar at 75:25 (169.78 mg kg^−1^ DM), increased numerically to 182.71 mg kg^−1^ DM at 50:50, and decreased to 161.24 mg kg^−1^ DM at 25:75. The 50:50 ratio resulted in significantly higher total β-carotene content than the 25:75 ratio, whereas neither treatment differed significantly from the control or 75:25 ratio. ‘Genovese’ had significantly higher total β-carotene content than ‘Sweet basil’ in monoculture, whereas no cultivar differences were detected at any of the three intercropping ratios.

Significant cultivar × intercropping ratio interactions were detected for several individual compounds, including 9-cis neoxanthin, trans-violaxanthin, trans-neoxanthin, 13-cis lutein, 13′-cis lutein, trans-lutein, 9-cis lutein, 7-cis lutein, 13-cis β-carotene, 13′-cis β-carotene, and trans-β-carotene (Table S1). These interactions indicate that the response of the respective carotenoids to intercropping differed between the two basil cultivars. In contrast, no significant cultivar × intercropping ratio interaction was detected for 15-cis lutein, trans-zeaxanthin, β-cryptoxanthin, α-carotene, 9-cis β-carotene, or 9′-cis β-carotene. Responses to intercropping therefore varied among individual carotenoids and, for several compounds, depended on basil cultivar. Significant cultivar × intercropping ratio interactions were further examined using simple-effect comparisons. Treatment-combination means and Tukey-adjusted comparisons for selected individual carotenoids are presented in Table S2, allowing a more detailed evaluation of cultivar-specific responses to intercropping.

In contrast to carotenoids, tocopherol content generally increased under intercropping. Total tocopherol content was significantly affected by cultivar (*p* = 0.0038) and intercropping ratio (*p* = 0.0043), whereas the cultivar × intercropping ratio interaction was not significant (*p* = 0.3240) (Table 3). Averaged across intercropping ratios, ‘Genovese’ had higher total tocopherol content than ‘Sweet basil’ (4690.75 vs. 4337.02 mg kg^−1^ DM). When averaged across cultivars, total tocopherol content was significantly higher at the 75:25 and 25:75 ratios (4760.09 and 4727.386 mg kg^−1^ DM, respectively) than in the control (4208.37 mg kg^−1^ DM). The 50:50 ratio showed an intermediate value (4359.70 mg kg^−1^ DM) and did not differ significantly from either the control or the other intercropping ratios.

**Figure 4:**
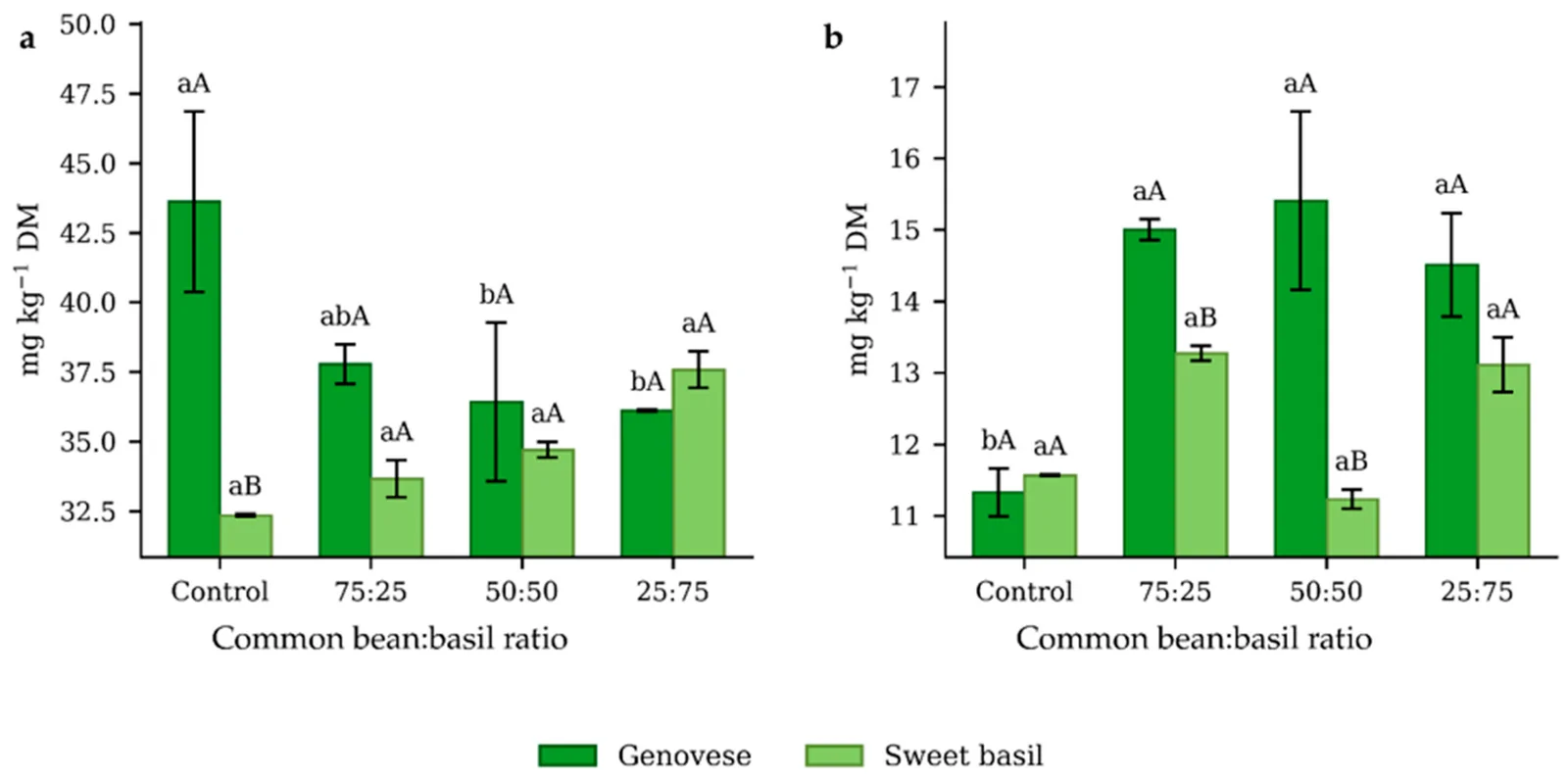
Significant cultivar × intercropping ratio interactions for tocopherols: (**a**) γ-tocopherol and (**b**) δ-tocopherol in basil leaves. Bars represent LSMeans ± SE calculated from individual plot observations (*n* = 3). Lowercase letters indicate significant differences among intercropping ratios within a cultivar, and uppercase letters indicate significant differences between cultivars within the same intercropping ratio, based on Tukey-adjusted comparisons of least-squares means (*p* < 0.05).

The response of total tocopherols was mainly associated with α-tocopherol, the predominant tocopherol, which was significantly affected by both cultivar (*p* = 0.0047) and intercropping ratio (*p* = 0.0045), without a significant interaction between the two factors (*p* = 0.3537; Table 3). When averaged across intercropping ratios, α-tocopherol content was higher in ‘Genovese’ than in ‘Sweet basil’ (4638.20 vs. 4290.15 mg kg^−1^ DM). When averaged across cultivars, α-tocopherol content was significantly higher at the 75:25 and 25:75 ratios (4710.22 and 4676.72 mg kg^−1^ DM, respectively) than in the control (4158.94 mg kg^−1^ DM), whereas the 50:50 ratio (4310.82 mg kg^−1^ DM) was intermediate. A significant cultivar × intercropping ratio interaction was detected for γ-tocopherol (*p* = 0.0108), although the main effect of intercropping ratio was not significant (*p* = 0.4488; Table 3, Figure 4). In ‘Genovese’, γ-tocopherol content decreased from 43.61 mg kg^−1^ DM in monoculture to 36.43 and 36.11 mg kg^−1^ DM at the 50:50 and 25:75 ratios, respectively, both of which were significantly lower than the control; the value at 75:25 (37.78 mg kg^−1^ DM) did not differ significantly from the control or the other intercropping treatments. In ‘Sweet basil’, γ-tocopherol content ranged from 32.35 to 37.58 mg kg^−1^ DM, with no significant differences among intercropping ratios. ‘Genovese’ had significantly higher γ-tocopherol content than ‘Sweet basil’ in monoculture, whereas no cultivar differences were detected under intercropping. A significant cultivar × intercropping ratio interaction was detected for δ-tocopherol (*p* = 0.0076; Table 3, Figure 4). δ-tocopherol was significantly affected by both cultivar (*p* = 0.0003) and intercropping ratio (*p* = 0.0007). In ‘Genovese’, δ-tocopherol increased from 11.33 mg kg^−1^ DM in monoculture to 15.00, 15.41, and 14.51 mg kg^−1^ DM at the 75:25, 50:50, and 25:75 ratios, respectively; all three intercropping treatments were significantly higher than the control but did not differ from one another. In ‘Sweet basil’, δ-tocopherol content ranged from 11.23 to 13.27 mg kg^−1^ DM, with no significant differences among treatments. ‘Genovese’ had significantly higher δ-tocopherol content than ‘Sweet basil’ at the 75:25 and 50:50 ratios, whereas the cultivars did not differ significantly in monoculture or at the 25:75 ratio.

### 2.3. Relationships Between Mineral Composition and Antioxidant Compounds

**Figure 5:**
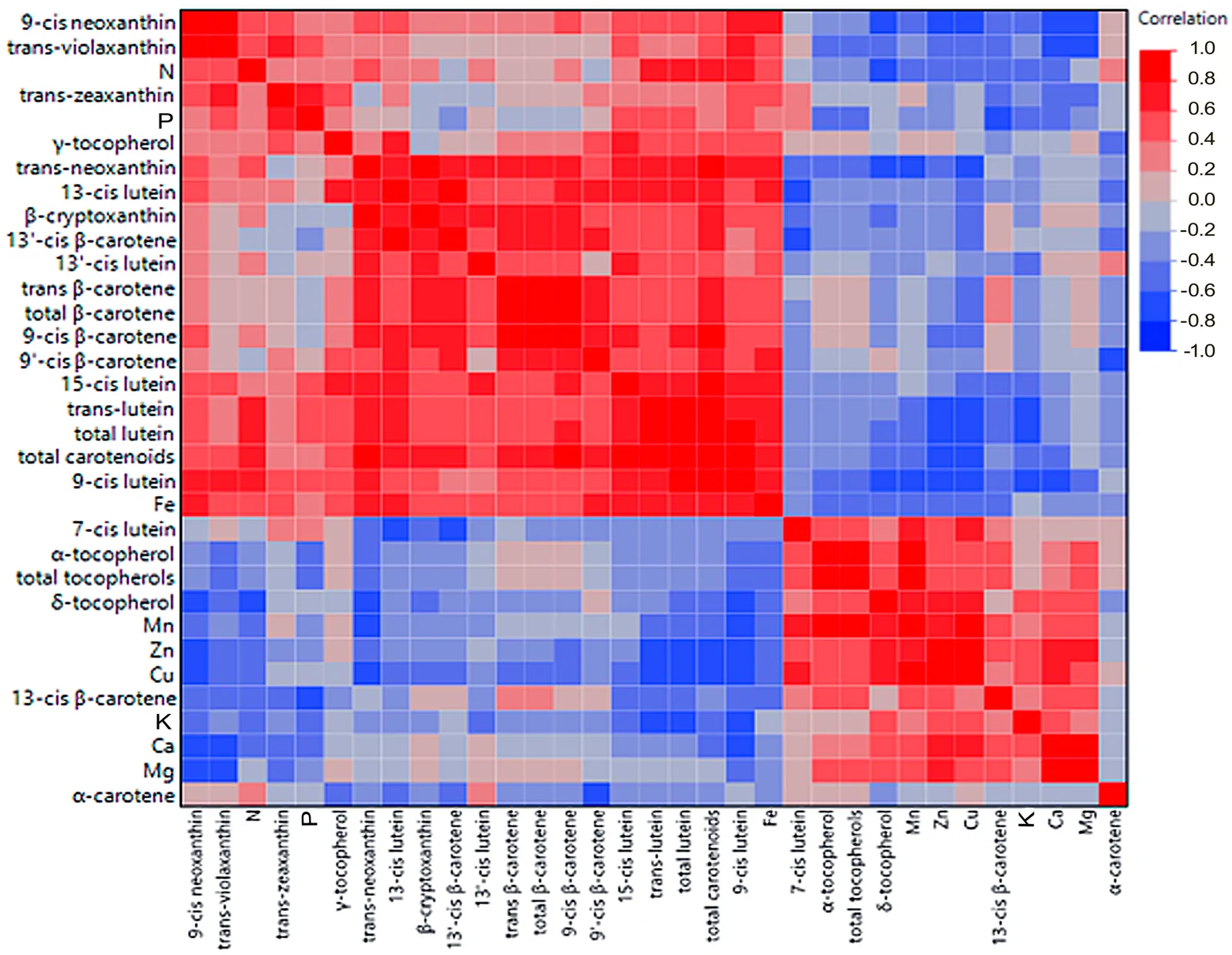
Pearson’s correlation matrix for minerals, carotenoids, and tocopherols in basil leaves based on plot-level observations (*n* = 24). The color scale represents Pearson’s correlation coefficient (r), ranging from −1 to +1.

Pearson’s correlation coefficients are presented in Supplementary Table S3, while the relationships among the analyzed compounds are illustrated in Figure 5. Among minerals, strong positive correlations were observed between Ca and Mg (r = 0.83), Zn and Cu (r = 0.91), Zn and Mn (r = 0.81), as well as Mn and Cu (r = 0.83). Calcium was also positively correlated with Zn (r = 0.74) and Cu (r = 0.67), while Mg was positively associated with Zn (r = 0.68). In contrast, N showed moderate to strong negative correlations with Zn (r = −0.57), Mn (r = −0.61), and Cu (r = −0.54), while Fe was negatively correlated with Zn (r = −0.52), Mn (r = −0.59), and Cu (r = −0.58). Potassium was negatively associated with several carotenoid compounds, particularly trans-lutein (r = −0.72), total lutein (r = −0.73), 9-cis lutein (r = −0.70), and total carotenoids (r = −0.69). Positive correlations were also observed among carotenoid compounds and their corresponding totals. Trans-lutein was highly correlated with total lutein (r = 0.997) and total carotenoids (r = 0.91), while trans-β-carotene strongly correlated with total β-carotene (r = 0.997), 9-cis β-carotene (r = 0.94), and total carotenoids (r = 0.75). Similarly, trans-neoxanthin showed strong positive associations with β-cryptoxanthin (r = 0.89), total carotenoids (r = 0.84), and total lutein (r = 0.79).

Several minerals were negatively associated with carotenoid content. Zn was negatively correlated with 9-cis neoxanthin (r = −0.83), 9-cis lutein (r = −0.80), total carotenoids (r = −0.61), and total lutein (r = −0.52). Cu showed a similar pattern, with negative correlations with 9-cis lutein (r = −0.81), trans-neoxanthin (r = −0.69), total carotenoids (r = −0.67), and total lutein (r = −0.60). Mn was negatively correlated with 9-cis lutein (r = −0.71), trans-neoxanthin (r = −0.64), and total lutein (r = −0.42). In contrast, Fe showed positive correlations with trans-lutein (r = 0.54), total lutein (r = 0.52), and 9-cis lutein (r = 0.56). Ca and Mg were negatively correlated with 9-cis neoxanthin (r = −0.73 and −0.84, respectively) and trans-violaxanthin (r = −0.68 and −0.81, respectively). 

Tocopherol homologs exhibited distinct correlation patterns. α-tocopherol was positively correlated with Mn (r = 0.69), Cu (r = 0.59), and Zn (r = 0.45), but negatively correlated with Fe (r = −0.64). Total tocopherols showed a similar pattern, with positive correlations with Mn (r = 0.69), Cu (r = 0.59), and Zn (r = 0.45), and a negative correlation with Fe (r = −0.64). γ-tocopherol was positively correlated with several carotenoids, including trans-lutein (r = 0.45), total lutein (r = 0.48), and total carotenoids (r = 0.52). In contrast, δ-tocopherol showed strong positive correlations with Cu (r = 0.82), Mn (r = 0.75), and Zn (r = 0.75), but was negatively associated with several carotenoid compounds, particularly 9-cis lutein (r = −0.74), 9-cis neoxanthin (r = −0.58), and trans-neoxanthin (r = −0.54). Thus, the relationships between mineral contents and lipophilic antioxidants differed between carotenoids and tocopherols and among individual compounds within these groups.

Of the 528 pairwise correlations, 240 were significant at *p* < 0.05 before adjustment, while 177 remained significant after applying the Benjamini–Hochberg FDR correction (q < 0.05).

### 2.4. Principal Component Analysis

**Figure 6:**
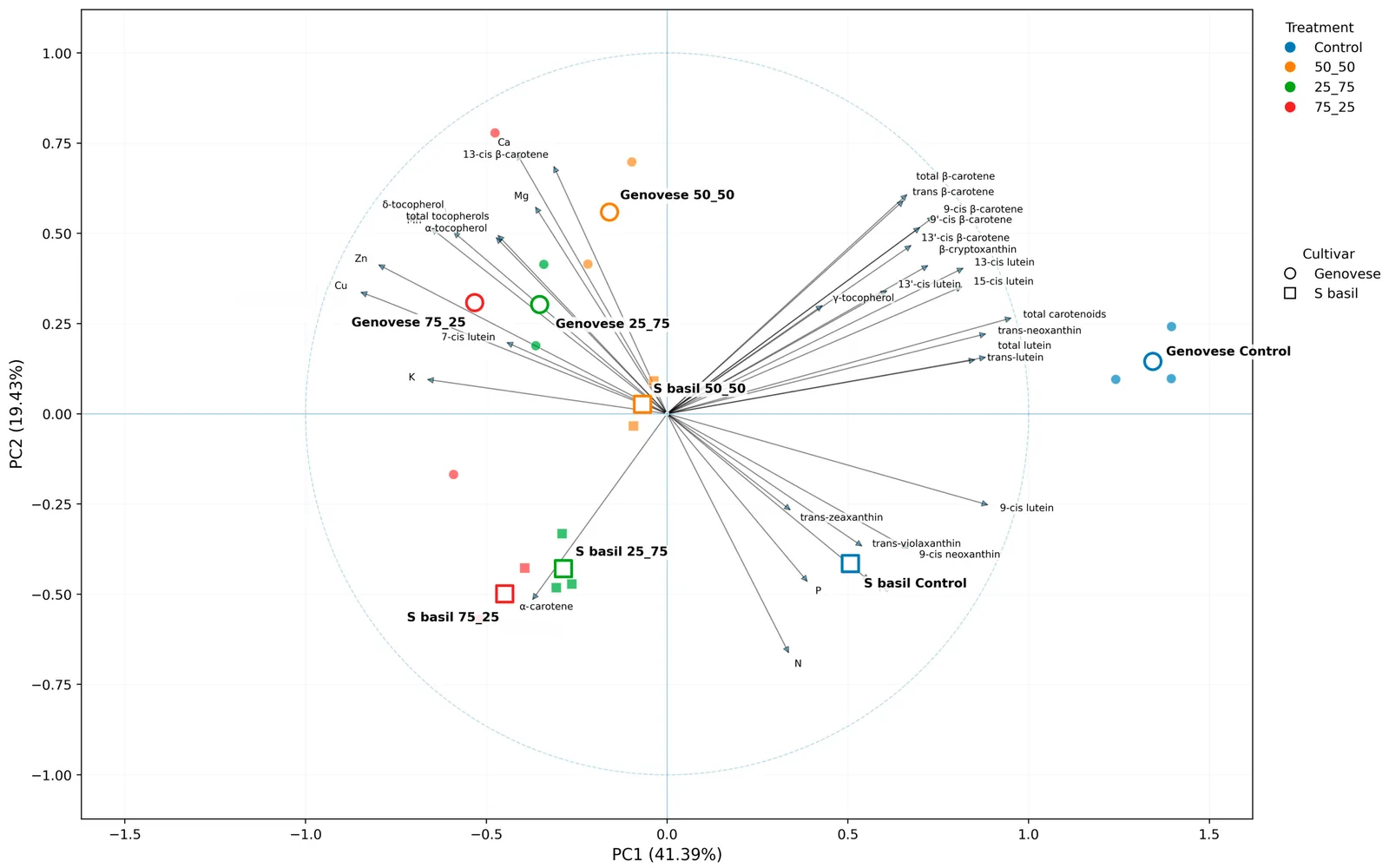
Principal component analysis (PCA) biplot showing relationships among minerals, carotenoids, and tocopherols and the distribution of basil samples according to cultivar and intercropping ratio.

Principal component analysis (PCA) (Figure 6) was performed to evaluate the overall variability among the analyzed samples and to identify the variables contributing most strongly to sample differentiation. Prior to analysis, all variables were standardized to unit variance and zero mean to account for differences in measurement scales.

The first two principal components explained 60.82% of the total variance in the dataset, with PC1 accounting for 41.39% and PC2 for 19.43% of the variance.

PC1 was mainly associated with carotenoid composition, particularly total carotenoids, total lutein, trans-lutein, 9-cis lutein, trans-neoxanthin, and several lutein isomers. In addition, Cu content showed a strong contribution to this component. The loading pattern indicated that carotenoid variables and selected micronutrients contributed in opposite directions along PC1, consistent with the negative associations between Zn and Cu and several carotenoid variables identified by Pearson’s correlation analysis.

PC2 was mainly influenced by mineral composition and β-carotene derivatives. Ca, Mg, N, total β-carotene, trans β-carotene, 13-cis β-carotene, and α-carotene contributed most to this component.

The PCA score plot showed a pattern of separation according to both cultivar and intercropping ratio. Monocropped ‘Genovese’ samples were positioned on the positive side of PC1, consistent with their distinct carotenoid profile, whereas monocropped ‘Sweet basil’ samples were characterized by positive PC1 and negative PC2 scores. Intercropped samples were mainly distributed toward negative PC1 scores, with additional separation along PC2 depending on cultivar and intercropping ratio.

The distribution of samples along the first two principal components reflected differences in both cultivar and intercropping ratio. Carotenoid variables, especially those related to lutein and β-carotene, together with Ca, Mg, Zn, Cu, Mn, and N, contributed markedly to this separation. The positions of Zn and Cu relate to several carotenoid variables along PC1 also agreed with the negative correlations observed in the Pearson analysis.

An additional PCA (not shown here) excluding the aggregate variables (total carotenoids, total lutein, total β-carotene, and total tocopherols) showed a similar overall pattern of sample distribution. In this analysis, PC1 and PC2 explained 39.46% and 19.75% of the total variance, respectively, accounting for 59.21% cumulatively. Sample scores were highly correlated with those obtained from the original PCA (r = 0.994 for PC1 and r = 0.984 for PC2), indicating that the main patterns of variation were largely maintained after removing the aggregate variables.

## 3. Discussion

Intercropping with common bean modified both the mineral and lipophilic antioxidant composition of basil leaves and, for several traits, the response depended on basil cultivar. Intercropping increased the contents of several mineral elements in a cultivar- and ratio-dependent manner, particularly Zn, Mn, Cu, Ca, Mg, and K, whereas N, P, and Fe decreased in some treatments. Total carotenoids and total lutein generally decreased, while total tocopherols increased in two intercropping treatments when averaged across cultivars. These responses differed among compositional traits and, for several traits, between cultivars.

### 3.1. Mineral Composition Under Different Intercropping Treatments

The increases in several mineral elements observed under basil–common bean intercropping in the present study are consistent with the findings of Rezaei-Chiyaneh et al. [9], who studied sweet basil–common bean intercropping at different planting ratios. They reported improved uptake of several macro- and micronutrients, particularly when intercropping was combined with vermicompost. Their results show that basil–common bean associations can modify nutrient acquisition, although the magnitude of the response depends on management and planting ratio.

An important distinction, however, is that Rezaei-Chiyaneh et al. [9] evaluated nutrient uptake, whereas the present study determined mineral contents in basil leaf dry matter. Nutrient uptake per unit area is influenced by both tissue content and biomass production and therefore does not necessarily correspond to increased mineral content in edible plant tissues. This is relevant to the pronounced Zn response observed in ‘Genovese’ in the present study, where leaf Zn content increased from approximately 60 mg kg^−1^ DM in monoculture to 103–107 mg kg^−1^ DM under intercropping, corresponding to increases of approximately 72–77%.

Changes in mineral acquisition under legume-based intercropping may result from resource complementarity and interspecific facilitation. Duchene et al. [13] described these belowground interactions as important mechanisms underlying improved resource use in legume-based intercrops. These mechanisms have been particularly well studied for P, Fe, and Zn in cereal–legume systems. Xue et al. [14] concluded that both niche complementarity and interspecific facilitation contribute to improved nutrient acquisition in intercropping systems. Root-induced changes in the rhizosphere, including altered nutrient solubility and mobilization, can increase access to poorly available mineral pools. They also emphasized that increased nutrient acquisition does not necessarily lead to higher nutrient contents in harvested organs, because yield and nutrient translocation also influence final nutrient contents. This distinction may explain the element-specific responses observed in the present experiment. While intercropping markedly enhanced Zn, Mn, and Cu contents, particularly in ‘Genovese’, Fe did not show a comparable increase. When averaged across cultivars, Fe content was significantly lower at the 75:25 and 50:50 ratios than in the control, whereas the 25:75 ratio was intermediate. Thus, the effect of intercropping on mineral composition depended on both the element and the basil cultivar. Such differences may reflect variation in nutrient availability, uptake, transport, and internal allocation rather than a uniform response of all mineral elements [19].

Significant cultivar × intercropping ratio interactions were observed for K, Ca, Mg, Zn, Mn, and Cu. ‘Genovese’ exhibited large changes in several micronutrients, with Zn increasing by approximately 72–77%, Mn by up to 56%, and Cu by up to 53% relative to monoculture. Ca and Mg contents in ‘Genovese’ were also significantly higher at all three intercropping ratios than in monoculture, although the magnitude of the increase differed among ratios. In ‘Sweet basil’, the responses were less uniform: Ca increased significantly at the 50:50 ratio, whereas Mg decreased significantly at 75:25 and did not differ from the control at 50:50 or 25:75. The Zn response illustrates the cultivar dependence particularly clearly. Under monoculture, ‘Sweet basil’ contained significantly more Zn than ‘Genovese’, but under intercropping Zn was consistently higher in ‘Genovese’ than under monoculture, with no clear trend across the three intercropping ratios. In contrast, Zn content in ‘Sweet basil’ remained relatively stable across all intercropping ratios and close to the monoculture value. Consequently, ‘Genovese’ had significantly higher Zn content than ‘Sweet basil’ at all three intercropping ratios. These results indicate cultivar-dependent differences in Zn concentration under the investigated cropping arrangements. However, the underlying mechanisms remain unclear, as the observed differences may reflect both interspecific interactions and changes in planting density or biomass accumulation. The present findings on mineral content under intercropping indicate that cultivar selection should be considered when designing basil intercropping systems, because the same cropping arrangement may produce different mineral responses in different cultivars.

The increases in several mineral elements were not accompanied by an increase in leaf N content. Across cultivars, N content was significantly lower at the 75:25 and 50:50 ratios than in the control, while the 25:75 ratio showed an intermediate response. In addition, ‘Sweet basil’ had higher overall N content than ‘Genovese’, but the absence of a significant cultivar × intercropping ratio interaction indicates that treatment responses did not differ significantly between cultivars. This finding does not contradict the expected role of legumes in intercropping. Biological N_2_ fixation does not imply immediate transfer of fixed N to the companion crop during the same growing season. Benefits may instead arise from complementary use of soil and atmospheric N and improved system-level N-use efficiency [13]. The absence of a consistent increase in basil N content therefore suggests that the pronounced increases in Zn, Mn, Cu, Ca, and Mg were not simply associated with higher leaf N content. Importantly, intercropping was not associated with uniformly higher mineral concentrations. In addition to the increases observed for certain elements, N and Fe concentrations were significantly lower at the 75:25 and 50:50 ratios than under sole cropping, while reductions in P were also observed. These results indicate that the effects of the investigated cropping arrangements on basil mineral composition were element-specific and should not be interpreted as an overall improvement in mineral nutritional quality.

### 3.2. Intercropping Effects on Basil Lipophilic Antioxidants

Previous studies have shown that cropping patterns can modify the phytochemical composition of basil. Kordi et al. [20], studying additive intercropping of sweet basil with forage maize (*Zea mays* L.), showed that intercropping and N2-fixing bacteria affected essential oil yield and composition. More recently, Ebrahimi et al. [21] reported that basil–fenugreek (*Trigonella foenum-graecum* L.) intercropping modified both essential oil yield and the relative abundance of individual essential oil constituents, showing that legume-based intercropping can also affect basil essential oil composition. Other intercropping systems have also shown compound-specific changes, including changes in flavonoid composition in lupin-barley mixtures [22]. These studies indicate that different groups of phytochemicals may respond differently to intercropping. Similarly, Kaur et al. [23] showed that co-cultivation of sweet basil with food crops affected basil productivity and essential oil content, indicating that intercropping outcomes depend strongly on species combination and planting ratio. Such compound-specific responses were also observed in the present study, where carotenoids and tocopherols responded differently to common bean intercropping.

#### 3.2.1. Carotenoid Response

In the present study, total carotenoids and particularly total lutein decreased under intercropping in both basil cultivars. The response was strongly cultivar dependent, as indicated by significant cultivar × intercropping ratio interactions for total carotenoids, total lutein, total β-carotene, and several individual carotenoids. The magnitude of the reduction in total carotenoids and total lutein varied among intercropping ratios and between cultivars. This agrees with the considerable variation in lutein, β-carotene, and total carotenoid content reported among basil genotypes grown under different environmental conditions [11]. Information on carotenoid composition in basil–legume intercropping systems remain scarce. The present study provides information on the responses of individual lutein, neoxanthin, zeaxanthin, and β-carotene fractions to common bean intercropping.

Reduced carotenoid content, however, does not appear to be a general response to intercropping. In some other species combinations, intercropping has produced neutral or positive responses. For example, Somarin et al. [24] reported higher carotenoid contents in camelina (*Camelina sativa* L. Crantz) intercropped with chickpea (*Cicer arietinum* L.) or lentil (*Lens culinaris* Medik.). Compared with those findings, the response observed in basil suggests that the effect of intercropping on carotenoids may vary with species combination, genotype, environmental conditions, and planting ratio.

The lower carotenoid contents observed under intercropping may be related to changes in the growing environment created by the associated crop. Differences in light availability, canopy structure, nutrient supply, and interspecific competition can alter resource availability in intercropping systems [13]. Carotenoids are closely associated with photosynthetic function and photoprotection, and changes in the plant growing environment may therefore affect their accumulation [18]. However, these factors were not measured in the present study, and their contribution to the observed carotenoid response cannot be determined directly.

In the present study, the response also differed among carotenoids. Lutein showed the clearest decline, whereas β-carotene exhibited a less uniform response. In ‘Genovese’, total β-carotene decreased significantly under all three intercropping ratios compared with monoculture, whereas in ‘Sweet basil’ its response was less consistent. However, total β-carotene at the 50:50 ratio was significantly higher than at the 25:75 ratio in ‘Sweet basil’. Thus, intercropping did not result in a uniform decline across all carotenoid compounds. Moreover, the significant cultivar × intercropping interactions observed for several individual carotenoid isomers indicate that their responses depended on both cultivar and intercropping ratio. The analytical procedure should also be considered when interpreting these results. Because recovery through the complete drying, saponification, and extraction procedure was not determined, the absolute carotenoid contents should be interpreted with some caution. However, all experimental samples were processed using the same procedure, supporting direct comparisons among cultivars and intercropping treatments within the present study.

#### 3.2.2. Tocopherol Responses

In contrast to carotenoids, total tocopherol content generally increased under intercropping, with significantly higher values at the 75:25 and 25:75 ratios than in monoculture when averaged across cultivars; α-tocopherol accounted for most of the total tocopherol content. Both total tocopherols and α-tocopherol were significantly affected by cultivar and intercropping ratio, whereas their cultivar × intercropping ratio interactions were not significant. The absence of significant interactions indicates no evidence that the effects of intercropping ratio on total tocopherol and α-tocopherol contents differed between the two cultivars. A similar increase in tocopherols has been reported in another legume-based intercropping system. Somarin et al. [24], for example, reported significantly higher total tocopherol contents in camelina intercropped with chickpea or lentil compared with sole cropping. In that study, however, carotenoids also increased, unlike the response observed in the present study. This comparison suggests that carotenoids and tocopherols do not necessarily respond in parallel to intercropping.

Individual tocopherol homologs also responded differently. α-tocopherol largely accounted for the changes in total tocopherol content, while the minor tocopherol homologs showed different response patterns. In particular, significant cultivar × intercropping ratio interactions were observed for both γ- and δ-tocopherol, indicating cultivar-specific responses of these minor homologs. The predominance of α-tocopherol is typical of photosynthetic tissues, where it plays an important role in protecting chloroplast membranes against oxidative damage, particularly by quenching singlet oxygen and limiting lipid peroxidation [18,25].

Tocopherol content in basil also appears to be responsive to growing conditions. Sgherri et al. [8] reported differences in vitamin E content between basil grown in soil and under hydroponic conditions, together with changes in other antioxidant compounds. Although the cultivation system in that study differed from the present experiment, these findings indicate that the tocopherol pool of basil leaves can respond to changes in the growing environment.

The opposite directions of the changes in carotenoid and tocopherol concentrations should not be interpreted as evidence of physiological compensation. The present measurements establish differences in the concentrations of these antioxidant groups, but not the processes responsible for them. Measurements of photosynthetic performance, oxidative status, and related metabolic pathways would be needed to investigate whether the observed changes are functionally connected.

The decrease in carotenoids accompanied by the increase in total tocopherol and α-tocopherol contents at intercropping ratios indicates contrasting changes in the concentrations of these two groups of lipophilic antioxidants. Although carotenoids and tocopherols both contribute to the protection of photosynthetic tissues [18,25], the observed differences do not demonstrate a compensatory relationship between them. As oxidative status, photosynthetic performance, and the regulation of carotenoid and tocopherol metabolism were not investigated, the mechanisms underlying these responses remain unclear. Therefore, the results are interpreted as changes in the lipophilic antioxidant composition of basil leaves rather than evidence of physiological compensation.

#### 3.2.3. Relationships Between Mineral Composition and Lipophilic Antioxidants

The simultaneous changes in mineral and antioxidant composition suggest a possible link between micronutrient status and the observed phytochemical response. Zn, Mn, and Cu have important roles in photosynthetic function and antioxidant metabolism as components or cofactors of proteins and enzymes involved in cellular redox regulation [16,26,27]. The marked enrichment of these elements, particularly in ‘Genovese’, was accompanied by changes in carotenoid and tocopherol concentrations, although the mechanisms underlying these relationships remain unclear. Recent biofortification experiments in basil also showed that changes in micronutrient status can be accompanied by changes in carotenoid accumulation and antioxidant enzyme activity [28]. More broadly, micronutrient homeostasis is closely connected with photosynthesis, ROS metabolism, and antioxidant defense [16,29].

The correlation analysis was consistent with this association, although relationships differed among antioxidant groups. Zn, Mn, and Cu, which increased under intercropping, particularly in ‘Genovese’, were negatively correlated with several carotenoid compounds. Zn and Cu showed negative correlations with 9-cis lutein, while both elements were also negatively associated with total carotenoids and/or total lutein. In contrast, Zn, Mn, and Cu were positively associated with α-tocopherol and total tocopherols, with correlations with Mn reaching r = 0.69. δ-tocopherol showed positive correlations with Cu (r = 0.82), Mn (r = 0.75), and Zn (r = 0.75), while being negatively associated with several carotenoid compounds. Comparable genotype-dependent relationships between mineral status and carotenoid accumulation have been reported in basil under Zn biofortification and different light environments [30,31]. However, other studies have reported simultaneous increases in mineral enrichment and carotenoids, indicating that the direction of these relationships depends on the element, genotype, and growing conditions [28]. Different responses of carotenoids and tocopherols to changes in mineral and redox status have also been reported under metal-induced stress, where α-tocopherol increased while another antioxidant group remained unchanged or declined [32]. This pattern is consistent with the contrasting carotenoid and tocopherol responses observed in the present study, although the observed associations do not provide evidence of physiological adjustments or direct relationships between mineral status and antioxidant metabolism.

PCA showed a similar overall pattern of relationships among mineral and antioxidant variables. Along PC1, the major carotenoid variables, including total carotenoids, total lutein, trans-lutein, and 9-cis lutein, loaded in the opposite direction to Zn and Cu. This pattern was consistent with the negative associations identified by Pearson’s correlation analysis. PC2 further reflected variation involving Ca, Mg, N, β-carotene derivatives, Mn, and tocopherols. Thus, variation in both mineral and antioxidant composition contributed to the multivariate distribution of the samples across the first two principal components. However, PC1 and PC2 together explained 60.82% of the total variance, indicating that a substantial proportion of the variability (39.18%) was captured by subsequent components. Therefore, the PCA biplot should be interpreted as a representation of the main patterns of variation rather than a complete description of the relationships among the analyzed traits and samples.

Furthermore, including total carotenoids and other aggregate variables together with their constituent compounds may have increased the contribution of carotenoid-related variables to the PCA. However, the additional analysis excluding these variables showed that the main patterns of sample distribution were largely maintained, indicating that the overall results were not substantially affected by this redundancy.

These associations do not establish causation. The experiment showed that mineral contents and lipophilic antioxidants changed concurrently across cultivar and intercropping treatments, but it does not establish that changes in mineral content directly caused the observed changes in carotenoid or tocopherol accumulation. The observed relationships may also partly reflect common responses of these traits to cultivar and intercropping ratio. Therefore, the negative associations between several micronutrients and carotenoids and their positive associations with tocopherols should be interpreted as associated changes in leaf composition rather than evidence of direct metabolic regulation.

Because the experiment was conducted at one location during a single growing season, the stability of the cultivar-specific responses across environments remains to be evaluated. In addition, biomass production, yield, canopy light conditions, rhizosphere nutrient availability, and physiological indicators of oxidative status were not measured, limiting conclusions about the mechanisms underlying the observed compositional changes. The observed differences in leaf mineral contents may also reflect differences in biomass accumulation and cannot be used to infer nutrient uptake per unit area. The stability of these cultivar-dependent responses and their underlying mechanisms require further investigation.

## 4. Materials and Methods

### 4.1. Plant Material and Experimental Conditions

Two basil cultivars, ‘Genovese’ (MAP00294) and ‘Sweet basil’ (MAP00414), were obtained from the Collection of Medicinal and Aromatic Plants of the National Plant Gene Bank of the Republic of Croatia (https://cpgrd.hapih.hr/), maintained by the Department of Plant Biodiversity, University of Zagreb Faculty of Agriculture, Croatia. Common bean seeds of the morphotype ‘Biser’, bush/dwarf type (market class Navy bean) were obtained from the Collection of the Centre of Excellence for Biodiversity and Molecular Plant Breeding (CoE CroP-BioDiv).

The field experiment was conducted at the Maksimir experimental station, University of Zagreb Faculty of Agriculture (45°49′ N, 16°20′ E). The experimental soil was classified as Cambisol according to the World Reference Base for Soil Resources [33]. General soil properties are shown in Table 4.

The experiment was arranged as a randomized complete block design with three replications, with two basil cultivars and four cropping treatments. The cropping treatments consisted of sole basil cultivation (control) and basil–common bean intercropping at common bean:basil proportions of 25:75, 50:50, and 75:25. Each plot contained four rows, each 4 m long and spaced 30 cm apart. In each intercropped row, common bean occupied 1, 2, or 3 m and basil occupied the remaining 3, 2, or 1 m, respectively. These proportions describe the lengths allocated to each species, not an independently controlled plant-density factor. Bean was sown at 2 cm within-row spacing and basil transplanted at 25 cm between plants. Thus, increasing the bean proportion also reduced the number of basil plants per plot.

Common bean seeds were sown on 5 May 2025, whereas uniformly developed basil seedlings were transplanted on 27 May 2025. Prior to transplanting, basil seedlings were produced in a greenhouse in polystyrene trays (52 × 32 cm, 60 cells) filled with a commercial growing substrate and maintained at a mean daily temperature of 20 °C.

Crop management followed conventional agricultural practices. NPK 7-20-30 fertilizer was applied once at the beginning of the growing season at a rate of 150 kg ha^−1^. Weeds were controlled mechanically. No irrigation was applied as precipitation during the experimental period was sufficient to meet crop water requirements.

Basil leaves were harvested on 22 July 2025 at the full flowering stage. Leaves collected from the two inner rows of each experimental plot were pooled to obtain one representative composite sample per plot, dried in a forced-air dryer at 38 °C, and homogenized for chemical analyses.

### 4.2. Determination of Mineral Contents

Dried basil leaf samples were homogenized, and each sample was analyzed in triplicate. The mean of the three analytical determinations was used as the value for each experimental plot in the statistical analysis. Total N content was determined using the Kjeldahl method according to AOAC procedures [34]. Following microwave-assisted acid digestion with concentrated HNO_3_ (MILESTONE 1200 Mega Microwave Digester, Milestone S.r.l., Milan, Italy), P content was measured spectrophotometrically, K by flame photometry, and Ca, Mg, Fe, Zn, Mn, and Cu by atomic absorption spectrophotometry (AAS), following AOAC [34] procedures.

### 4.3. Carotenoids and Tocopherols

Carotenoids and tocopherols were extracted simultaneously from dried basil leaves using a procedure adapted from Montoya-Arroyo et al. [35]. Briefly, 0.10 g of homogenized leaf material was combined with 2 mL of ethanol containing 1% (*w*/*v*) ascorbic acid, 0.90 mL of deionized water, and 0.60 mL of saturated KOH solution. The mixture was saponified for 30 min at 70 °C under continuous agitation, then cooled on ice. Butylated hydroxytoluene (BHT; 25 µL, 1 mg mL^−1^ in ethanol) was added, followed by 0.60 mL of glacial acetic acid for neutralization. Lipophilic compounds were recovered with successive extractions with HPLC-grade n-hexane until a colorless extract was obtained. The combined organic phases were evaporated to dryness under reduced pressure and protected from light. Before HPLC analysis, the residue was reconstituted in methanol/MTBE (60:40, *v*/*v*), according to the solvent conditions used for green plant tissues by Gupta et al. [36].

Carotenoids and tocopherols were determined in a single chromatographic run using a SpectraSystem HPLC system (Thermo Separation Products, Waltham, MA, USA) equipped with a quaternary pump, autosampler, UV/Vis detector, and fluorescence detector. Chromatographic separation was performed according to Gupta et al. [36] on a C30 reversed-phase column (3 µm, 250 × 4.6 mm) fitted with a C30 guard column. The mobile phases consisted of (A) methanol/water (98:2, *v*/*v*), (B) methanol/water (95:5, *v*/*v*), and (C) methyl tert-butyl ether (MTBE). The gradient started at 80% A and 20% C, changed linearly to 60% A and 40% C within 2 min, followed by 60% B and 40% C and a gradual increase to 100% C at 12 min; the initial conditions were restored at 13 min and maintained for column re-equilibration. The column temperature was maintained at 20 °C. Carotenoids were monitored by UV/Vis detection at 450 nm, while tocopherols were recorded simultaneously by fluorescence detection at excitation and emission wavelengths of 290 and 330 nm, respectively.

Quantification was performed by external calibration using commercially available carotenoid and tocopherol standards. Trans-carotenoids were identified by comparison of their retention times with those of commercially available authentic standards, whereas cis-isomers were tentatively identified based on their retention behavior relative to the cis-isomers reported by Gupta et al. [36]. Gupta et al. [36] used this approach because of the similarity in extinction coefficients between the respective *trans*- and *cis*-isomers. Because *cis*-isomer standards and isomer-specific response factors were not available, the contents of individual *cis*-isomers should be regarded as semi-quantitative estimates. For carotenoid standards, LOD ranged from 0.075 to 0.145 µg mL^−1^ and LOQ from 0.229 to 0.439 µg mL^−1^, whereas for tocopherols the corresponding ranges were 0.125–0.134 and 0.380–0.405 µg mL^−1^, respectively. Standard-solution accuracy was evaluated by analysis of standard solutions of known concentration and ranged from 98.90 to 102.88%. Repeatability of the analytical procedure was assessed by six replicate determinations of the same homogenized basil sample. Relative standard deviations ranged from 1.34 to 5.91% among the quantified carotenoids and tocopherols. Total carotenoid and total tocopherol contents were calculated as the sums of the respective individually quantified compounds. Results were expressed on a dry matter basis.

### 4.4. Data Analysis

All statistical analyses were performed using JMP Pro 18 (SAS Institute Inc., Cary, NC, USA). Data were analyzed as a randomized complete block design with three blocks. Basil cultivar (C; ‘Genovese’ and ‘Sweet basil’), intercropping ratio (I; sole cropping and three basil–common bean intercropping ratios), and their interaction (C × I) were included as fixed effects, whereas block was included as a random effect. Variance components associated with the random block effect were estimated using restricted maximum likelihood (REML). Fixed effects were evaluated using F-tests, with numerator and denominator degrees of freedom obtained from the fitted mixed model. Model assumptions were assessed by examining residual diagnostic plots for deviations from normality and homogeneity of variance. The experimental plot was considered the experimental unit (*n* = 3). Least-squares means (LSMeans) were compared using Tukey-adjusted pairwise comparisons at *p* < 0.05. When a significant C × I interaction was detected, intercropping ratios were compared within each cultivar, and cultivars were compared within each intercropping ratio. These comparisons included differences among intercropping ratios within each cultivar and differences between cultivars within each intercropping ratio. Tukey’s adjustment was applied across the eight cultivar × intercropping ratio combinations. Main effect results and treatment-combination means are presented as LSMeans with their model-based standard errors (SE).

Pearson’s correlation analysis was performed to assess relationships among mineral contents, individual and total carotenoids, and tocopherols. Correlation coefficients (r) were calculated using plot-level observations (*n* = 24). To account for multiple testing, *p*-values from the 528 pairwise correlations among the 33 variables were adjusted using the Benjamini–Hochberg false discovery rate (FDR) procedure. Correlations with FDR-adjusted *p*-values below 0.05 were considered statistically significant.

Principal component analysis (PCA) was performed using the same plot-level observations to examine multivariate relationships among mineral and antioxidant traits and to visualize variation associated with cultivar and intercropping ratio. Prior to PCA, variables were standardized to zero mean and unit variance (z-scores). The first two principal components were used to describe sample distribution and the variables contributing most strongly to differentiation among samples. To assess the influence of mathematically related variables, an additional PCA was performed after excluding total carotenoids, total lutein, total β-carotene, and total tocopherols. The similarity between the original and reduced PCA solutions was evaluated by comparing the explained variance and the sample scores of the first two principal components. Both analyses were considered exploratory.

## 5. Conclusions

The effects of basil–common bean intercropping on leaf composition differed among mineral elements and lipophilic antioxidants. Under the investigated growing conditions, intercropping was associated with higher concentrations of several mineral elements in specific cultivar–treatment combinations, whereas N, P, and Fe generally decreased under certain intercropping ratios. Total carotenoids and total lutein decreased in both cultivars, while total tocopherols increased at the 75:25 and 25:75 ratios when averaged across cultivars. These results indicate that changes in basil leaf composition were not uniformly beneficial and depended on the compounds analyzed.

Significant cultivar × intercropping ratio interactions for K, Ca, Mg, Zn, Mn, and Cu showed that mineral responses differed between the two cultivars. This was particularly evident for Zn, which showed consistently higher content under all intercropping ratios in ‘Genovese’ than under monoculture, without a clear trend across the three ratios, but remained relatively stable in ‘Sweet basil’. Carotenoid composition was also cultivar dependent, with total carotenoids and total lutein decreasing under intercropping in both cultivars, while β-carotene showed a less uniform response. In contrast, total tocopherol and α-tocopherol contents were affected by cultivar and intercropping ratio without significant interactions between these factors, whereas γ- and δ-tocopherol showed cultivar-dependent responses. The opposite trends observed for carotenoids and tocopherols suggest different responses of these antioxidant groups to the investigated treatments, but do not indicate a compensatory relationship between them. Correlation analysis and PCA revealed associations among mineral elements and lipophilic antioxidants, particularly between Zn, Mn, Cu, and several carotenoid and tocopherol compounds. However, these associations may partly reflect differences among cultivars and treatments and cannot be used to infer direct physiological relationships.

The combined analysis of mineral composition, individual carotenoids, and tocopherols showed that increases in some components of basil leaf composition can occur together with reductions in others. Cultivar and intercropping ratio should therefore be considered when evaluating compositional responses to intercropping, particularly for traits showing significant cultivar × intercropping ratio interactions. Evaluation of basil intercropping systems should include both mineral and phytochemical composition rather than relying on a single group of quality traits. However, as the intercropping treatments also involved changes in planting density, further studies including appropriate density controls and yield measurements are needed to distinguish the effects of interspecific interactions from those associated with plant density.

## Figures and Tables

**Table 1:** Effects of basil cultivar, intercropping ratio, and their interaction on mineral contents in basil leaves.

	N	P	K	Ca	Mg	Fe	Zn	Mn	Cu
	g kg^−1^ DM					mg kg^−1^ DM			
Cultivar									
‘Genovese’	36.64 b	3.60	24.60	48.38 a	7.09 a	1617.53 b	94.32 a	165.43 a	27.90 a
‘Sweet basil’	38.32 a	3.65	24.92	43.96 b	6.82 b	1734.07 a	85.20 b	142.91 b	25.26 b
SE	0.35	0.04	0.17	0.34	0.03	31.82	0.86	2.68	0.27
Intercropping ratio (common bean:basil)									
Control	38.67 a	3.79 a	23.15 b	42.84 b	6.69 c	1837.90 a	72.77 b	133.11 c	21.84 c
75:25	36.71 b	3.66 ab	25.13 a	44.67 b	6.74 c	1544.85 b	96.71 a	177.04 a	29.06 a
50:50	36.56 b	3.50 b	25.67 a	49.35 a	7.33 a	1634.00 b	95.75 a	151.36 b	26.62 b
25:75	38.00 ab	3.55 b	25.08 a	47.80 a	7.07 b	1686.44 ab	93.82 a	155.18 b	28.81 a
SE	0.45	0.05	0.22	0.48	0.05	44.99	1.22	3.32	0.38
*p*									
Cultivar (C)	0.0009	0.4148	0.1475	<0.0001	<0.0001	0.0214	<0.0001	<0.0001	<0.0001
Intercropping ratio (I)	0.0058	0.0090	<0.0001	<0.0001	<0.0001	0.0032	<0.0001	<0.0001	<0.0001
C × I	0.0528	0.0869	0.0077	0.0008	<0.0001	0.5397	<0.0001	<0.0001	<0.0001

Values are least-squares means (*n* = 12 for cultivar; *n* = 6 for intercropping ratio). SE, model-based standard error of the least-squares mean. Within a factor, means followed by different letters differ significantly (Tukey-adjusted comparisons, *p* < 0.05); letters are shown only for significant main effects.

**Table 2:** Effects of basil cultivar, intercropping ratio, and their interaction on major carotenoid contents in basil leaves.

	*trans*-Neoxanthin	*trans*-Violaxanthin	*trans*-Lutein	*trans*-Zeaxanthin	β-Cryptoxanthin	α-Carotene	*trans* β-Carotene	Total Lutein	Total β-Carotene	Total Carotenoids
	mg kg^−1^ DM									
Cultivar										
‘Genovese’	28.04 a	12.11	362.79 a	20.55	3.65 a	16.43 b	148.23 a	415.98 a	186.10 a	702.26 a
‘Sweet basil’	26.55 b	12.10	350.15 b	19.76	3.27 b	17.97 a	136.12 b	399.67 b	171.02 b	669.23 b
SE	0.19	0.15	2.18	0.33	0.07	0.58	2.33	2.16	3.00	3.07
Intercropping ratio (common bean: basil)										
Control	31.05 a	13.76 a	392.71 a	22.10 a	3.98 a	16.71	150.61 a	451.28 a	189.31 a	750.62 a
75:25	23.05 d	13.04 a	332.05 c	22.10 a	2.77 c	18.61	139.20 bc	380.04 c	173.93 bc	652.18 c
50:50	28.23 b	10.65 b	354.11 b	16.91 c	3.61 ab	15.97	146.74 ab	403.98 b	184.50 ab	680.85 b
25:75	26.84 c	10.96 b	347.03 b	19.50 b	3.48 b	17.52	132.14 c	396.00 b	166.49 c	659.33 c
SE	0.27	0.21	3.08	0.46	0.11	0.74	2.98	3.05	3.78	3.94
*p*										
Cultivar (C)	<0.0001	0.9430	0.0011	0.1104	0.0029	0.0343	0.0004	0.0001	0.0004	<0.0001
Intercropping ratio (I)	<0.0001	<0.0001	<0.0001	<0.0001	<0.0001	0.0681	0.0010	<0.0001	0.0009	<0.0001
C × I	<0.0001	0.0001	0.0007	0.1602	0.4056	0.0601	0.0051	0.0004	0.0094	<0.0001

Values are least-squares means (*n* = 12 for cultivar; *n* = 6 for intercropping ratio). SE, model-based standard error of the least-squares mean. Within a factor, means followed by different letters differ significantly (Tukey-adjusted comparisons, *p* < 0.05); letters are shown only for significant main effects.

**Table 3:** Effects of basil cultivar, intercropping ratio, and their interaction on tocopherol contents in basil leaves.

	α-Tocopherol	γ-Tocopherol	δ-Tocopherol	Total Tocopherols
	mg kg^−1^ DM			
Cultivar				
‘Genovese’	4638.20 a	38.48 a	14.06 a	4690.75 a
‘Sweet basil’	4290.15 b	34.58 b	12.30 b	4337.02 b
SE	73.43	0.82	0.30	73.69
Intercropping ratio (common bean:basil)				
Control	4158.94 b	37.98	11.45 b	4208.37 b
75:25	4710.22 a	35.73	14.14 a	4760.09 a
50:50	4310.82 ab	35.57	13.32 a	4359.70 ab
25:75	4676.72 a	36.85	13.81 a	4727.38 a
SE	103.85	1.16	0.40	104.21
*p*				
Cultivar (C)	0.0047	0.0046	0.0003	0.0044
Intercropping ratio (I)	0.0045	0.4488	0.0007	0.0045
C × I	0.3537	0.0108	0.0076	0.3503

Values are least-squares means (*n* = 12 for cultivar; *n* = 6 for intercropping ratio). SE, model-based standard error of the least-squares mean. Within a factor, means followed by different letters differ significantly (Tukey-adjusted comparisons, *p* < 0.05); letters are shown only for significant main effects.

**Table 4:** Physical and chemical properties of the soil from experimental field.

pH (H_2_O)	6.31
pH (KCl)	5.09
Organic matter (%)	0.89
Total nitrogen (%)	0.09
P_2_O_5_ (mg/100 g)	11.7
K_2_O (mg/100 g)	16.2
Clay (%)	15.4
Silt (%)	67.4
Sand (%)	17.2

## Data Availability

The data that support the findings of this study are available from the corresponding author, KCS, upon reasonable request.

## Supplementary Materials

The following supporting information can be downloaded at https://www.mdpi.com/article/10.3390/plants15213443/s1, Table S1: Main effects and ANOVA results for individual carotenoid compounds showing significant cultivar × intercropping ratio interactions Table S2: Cultivar × intercropping ratio interactions and Tukey-adjusted simple-effect comparisons for carotenoids in basil leaves, Table S3: Pearson’s correlation coefficients (r) and their statistical significance for mineral elements, carotenoids, and tocopherols in basil leaves, and Figure S1: Comparison of principal component analysis (PCA) based on (A) the original dataset comprising 33 mineral and antioxidant variables and (B) the reduced dataset comprising 29 variables after excluding total carotenoids, total lutein, total β-carotene, and total tocopherols.
