# Supplementary material for: Cultivar-Dependent Effects of Basil–Common Bean Intercropping on Mineral and Lipophilic Antioxidant Composition
Source: Plants (Basel). 2026 Nov 5;15(21):3443. doi: 10.3390/plants15213443 (PMC13766149; doi:10.3390/plants15213443)
Supplement: Supplementary file 1 [file plants-15-03443-s001.zip › Table S1.pdf]

Table S1. Main effects and ANOVA results for individual carotenoid compounds showing significant cultivar × intercropping ratio interactions.

**Carotenoid**	**Cultivar (C)** mg kg⁻¹ DM			**Intercropping ratio (common bean:basil; I)** mg kg⁻¹ DM					***p***		
	**'Genovese'**	**'Sweet basil'**	**SE**	**Control**	**75:25**	**50:50**	**25:75**	**SE**	**Cultivar**	**Ratio**	**C×I**
9-*cis* neoxanthin	18.49	18.81	0.14	20.47a	18.58b	17.00c	18.56b	0.20	0.1317	<0.0001	<0.0001
trans-violaxanthin	12.11	12.10	0.15	13.76a	13.04a	10.65b	10.96b	0.21	0.9430	<0.0001	0.0001
trans-neoxanthin	28.04a	26.55b	0.19	31.05a	23.05d	28.23b	26.84c	0.27	<0.0001	<0.0001	<0.0001
13-*cis* lutein	16.82a	15.64b	0.12	17.76a	15.08c	16.38b	15.70bc	0.17	<0.0001	<0.0001	0.0035
13'-*cis* lutein	9.08a	7.91b	0.16	9.53a	7.79b	8.45b	8.20b	0.22	0.0001	0.0005	0.0128
trans-lutein	362.79a	350.15b	2.18	392.71a	332.05c	354.11b	347.03b	3.08	0.0011	<0.0001	0.0007
9-*cis* lutein	8.67b	9.26a	0.15	12.74a	6.88c	7.78b	8.46b	0.22	0.0153	<0.0001	<0.0001
7-*cis* lutein	8.13a	7.60b	0.06	7.26c	9.00a	7.54bc	7.66b	0.09	<0.0001	<0.0001	<0.0001
13-*cis* β-carotene	4.85a	4.62b	0.09	4.37b	4.74ab	5.13a	4.69b	0.11	0.0344	0.0009	0.0138
13'-*cis* β-carotene	10.59a	9.62b	0.20	11.31a	9.02b	10.98a	9.10b	0.25	0.0006	<0.0001	0.0001
trans β-carotene	148.23a	136.12b	2.33	150.61a	139.20bc	146.74ab	132.14c	2.98	0.0004	0.0010	0.0051

Values are least-squares means. SE, model-based standard error of the least-squares mean. Different lowercase letters within a factor indicate significant differences according to Tukey-adjusted comparisons ($p < 0.05$); letters are shown only for significant main effects
