# Supplementary material for: Cultivar-Dependent Effects of Basil–Common Bean Intercropping on Mineral and Lipophilic Antioxidant Composition
Source: Plants (Basel). 2026 Nov 5;15(21):3443. doi: 10.3390/plants15213443 (PMC13766149; doi:10.3390/plants15213443)
Supplement: Supplementary file 1 [file plants-15-03443-s001.zip › Table S2.pdf]

Supplementary Table S2. Cultivar × intercropping ratio interactions and Tukey-adjusted simple-effect comparisons for carotenoids in basil leaves.

Cultivar × intercropping ratio (common bean:basil)	9-cis neoxanthin	trans-violaxanthin	trans-neoxanthin	13-cis lutein	13'-cis lutein	trans-lutein	9-cis lutein	7-cis lutein	13-cis β-carotene	13'-cis β-carotene	trans β-carotene
Genovese – Control	22.06 ± 0.28 a	14.59 ± 0.28 a	33.44 ± 0.37 a	18.53 ± 0.23 a	10.07 ± 0.31 a	398.80 ± 4.18 a	13.60 ± 0.30 a	7.74 ± 0.11 cd	4.52 ± 0.15 bc	12.28 ± 0.34 a	166.69 ± 3.98 a
Genovese – 75:25	17.11 ± 0.28 de	12.12 ± 0.28 cd	23.29 ± 0.37 d	15.13 ± 0.23 cd	8.21 ± 0.31 bcd	351.74 ± 4.18 b	7.38 ± 0.30 d	9.53 ± 0.11 a	5.05 ± 0.15 ab	8.49 ± 0.34 cd	142.97 ± 3.98 b
vs Genovese control (adjusted p)	<0.0001	0.0003	<0.0001	<0.0001	0.0124	<0.0001	<0.0001	<0.0001	0.2686	<0.0001	0.0117
Genovese – 50:50	16.31 ± 0.28 e	10.24 ± 0.28 e	28.15 ± 0.37 bc	16.84 ± 0.23 b	9.75 ± 0.31 ab	357.99 ± 4.18 b	6.24 ± 0.30 d	7.28 ± 0.11 de	4.93 ± 0.15 abc	11.15 ± 0.34 ab	147.82 ± 3.98 ab
vs Genovese control (adjusted p)	<0.0001	<0.0001	<0.0001	0.0016	0.9947	<0.0001	<0.0001	0.1592	0.5513	0.3109	0.0610
Genovese – 25:75	18.49 ± 0.28 c	11.50 ± 0.28 de	27.27 ± 0.37 bc	16.79 ± 0.23 b	8.30 ± 0.31 bcd	342.65 ± 4.18 b	7.45 ± 0.30 d	7.97 ± 0.11 bc	4.89 ± 0.15 abc	10.44 ± 0.34 b	135.44 ± 3.98 b
vs Genovese control (adjusted p)	<0.0001	<0.0001	<0.0001	0.0012	0.0189	<0.0001	<0.0001	0.8311	0.6626	0.0228	0.0009
Sweet basil – Control	18.87 ± 0.28 bc	12.93 ± 0.28 bc	28.65 ± 0.37 b	16.98 ± 0.23 b	8.98 ± 0.31 abc	386.62 ± 4.18 a	11.89 ± 0.30 b	6.78 ± 0.11 e	4.22 ± 0.15 c	10.35 ± 0.34 b	134.53 ± 3.98 b
Sweet basil – 75:25	20.06 ± 0.28 b	13.96 ± 0.28 ab	22.82 ± 0.37 d	15.04 ± 0.23 cd	7.38 ± 0.31 d	312.35 ± 4.18 c	6.38 ± 0.30 d	8.46 ± 0.11 b	4.42 ± 0.15 bc	9.55 ± 0.34 bc	135.43 ± 3.98 b
vs Sweet basil control (adjusted p)	0.1090	0.2325	<0.0001	0.0004	0.0373	<0.0001	<0.0001	<0.0001	0.9718	0.6948	1.0000
Sweet basil – 50:50	17.69 ± 0.28 cd	11.07 ± 0.28 de	28.32 ± 0.37 b	15.92 ± 0.23 bc	7.16 ± 0.31 d	350.22 ± 4.18 b	9.32 ± 0.30 c	7.80 ± 0.11 cd	5.34 ± 0.15 a	10.82 ± 0.34 ab	145.67 ± 3.98 b
vs Sweet basil control (adjusted p)	0.1071	0.0049	0.9978	0.0712	0.0146	0.0003	0.0003	0.0002	0.0015	0.9706	0.5218
Sweet basil – 25:75	18.62 ± 0.28 c	10.42 ± 0.28 e	26.41 ± 0.37 c	14.61 ± 0.23 d	8.10 ± 0.31 cd	351.41 ± 4.18 b	9.46 ± 0.30 c	7.35 ± 0.11 d	4.49 ± 0.15 bc	7.76 ± 0.34 d	128.84 ± 3.98 b
vs Sweet basil control (adjusted p)	0.9975	0.0002	0.0099	<0.0001	0.5215	0.0004	0.0006	0.0470	0.8862	0.0011	0.9659
Genovese vs Sweet basil – Control (adjusted p)	<0.0001	0.0135	<0.0001	0.0038	0.2864	0.4767	0.0153	0.0005	0.8256	0.0158	0.0007
Genovese vs Sweet basil – 75:25 (adjusted p)	<0.0001	0.0054	0.9817	1.0000	0.5864	0.0001	0.3051	0.0001	0.1261	0.3880	0.8706
Genovese vs Sweet basil – 50:50 (adjusted p)	0.0432	0.4661	1.0000	0.1528	0.0005	0.8802	<0.0001	0.0837	0.5632	0.9959	0.9999
Genovese vs Sweet basil – 25:75 (adjusted p)	0.9999	0.1922	0.7131	0.0001	0.9997	0.8063	0.0037	0.0242	0.5787	0.0008	0.9288

Values are least-squares means ± model-based SE (mg kg^{-1} DM). Different lowercase letters indicate Tukey groupings among all cultivar × treatment combinations for each compound ($p < 0.05$). Adjusted p-values refer to the indicated simple-effect comparisons; values below 0.05 indicate statistical significance. Control = sole basil cropping.
