# Supplementary material for: Cultivar-Dependent Effects of Basil–Common Bean Intercropping on Mineral and Lipophilic Antioxidant Composition
Source: Plants (Basel). 2026 Nov 5;15(21):3443. doi: 10.3390/plants15213443 (PMC13766149; doi:10.3390/plants15213443)
Supplement: Supplementary file 1 [file plants-15-03443-s001.zip › Table S3.pdf]

Table S3. Pearson’s correlation coefficients (r) and their statistical significance for mineral elements, carotenoids, and tocopherols in basil leaves.

	9-cis neoxanthin	trans-viol	trans-neo	15-cis lute	13-cis lute	13'-cis lut	trans-lute	9-cis lutei	trans-zeax	7-cis lutei	β-cryptox	13-cis β-ca	13'-cis β-c	α-caroten	trans β-ca	9-cis β-car	9'-cis β-ca	total carot	total lutei	total β-car	α-tocophe	γ-tocophe	δ-tocophe	total toco	N	P2O5	K2O	Ca	Mg	Fe	Zn	Mn	Cu
**9-cis neoxanthin**	1	0,819832	0,546569	0,441809	0,444325	0,2631	0,400713	0,783597	0,592649	-0,0767	0,251702	-0,43482	0,234685	0,040855	0,370287	0,414757	0,344364	0,583807	0,460062	0,359355	-0,26436	0,369525	-0,67025	-0,26235	0,559722	0,25662	-0,41897	-0,75549	-0,64982	0,66959	-0,64612	-0,49736	-0,61247
**trans-violaxanthin**	0,81983202	1	0,257052	0,500279	0,238236	0,170395	0,339867	0,653549	0,781419	0,127057	0,033854	-0,59121	0,018013	0,05332	0,17589	0,146083	0,16076	0,44717	0,39185	0,145968	-0,42363	0,303104	-0,55749	-0,42112	0,421141	0,585215	-0,39154	-0,72365	-0,75795	0,599319	-0,58454	-0,35928	-0,47641
**trans-neoxanthin**	0,546569331	0,257052	1	0,664301	0,772279	0,667215	0,705409	0,759253	-0,03096	-0,53749	0,827279	-0,14402	0,723589	-0,22607	0,628992	0,674685	0,531272	0,823499	0,743982	0,652022	-0,42183	0,337494	-0,60985	-0,41919	0,562107	0,06247	-0,39402	-0,19373	-0,05116	0,758095	-0,4838	-0,60167	-0,64674
**15-cis lutein**	0,441808586	0,500279	0,664301	1	0,741718	0,677737	0,746956	0,669797	0,386217	-0,26166	0,480394	-0,42451	0,521109	-0,33075	0,592082	0,60882	0,494037	0,822068	0,791152	0,588326	-0,21343	0,668265	-0,35369	-0,20689	0,392725	0,468988	-0,51946	-0,22523	-0,03994	0,651515	-0,33038	-0,19696	-0,48166
**13-cis lutein**	0,444324699	0,238236	0,772279	0,741718	1	0,466068	0,678906	0,565466	0,219079	-0,61615	0,63621	-0,34065	0,809493	-0,59069	0,5598	0,652612	0,73157	0,746385	0,705153	0,597993	-0,21974	0,608264	-0,20934	-0,21315	0,233039	0,051876	-0,25159	-0,13642	-0,07321	0,70823	-0,38844	-0,32969	-0,51796
**13'-cis lutein**	0,263100278	0,170395	0,667215	0,677737	0,466068	1	0,461396	0,345534	-0,12361	-0,35833	0,60789	-0,20147	0,414455	0,240615	0,485847	0,531906	0,15756	0,584353	0,501195	0,487261	-0,15447	0,146954	-0,35315	-0,15386	0,363116	0,070766	-0,42002	0,091324	0,194088	0,434987	-0,13703	-0,21332	-0,33524
**trans-lutein**	0,400713413	0,339867	0,705409	0,746956	0,678906	0,461396	1	0,772307	0,354516	-0,38759	0,556345	-0,47436	0,429918	-0,29625	0,474325	0,595695	0,567147	0,913241	0,995604	0,482894	-0,35833	0,487461	-0,37474	-0,35324	0,649548	0,565638	-0,62488	-0,33903	-0,15234	0,682021	-0,63635	-0,49282	-0,65277
**9-cis lutein**	0,783596689	0,653549	0,759253	0,669797	0,565466	0,345534	0,772307	1	0,423664	-0,24923	0,473606	-0,40577	0,377299	-0,17845	0,465895	0,504424	0,409146	0,829394	0,812188	0,461168	-0,42709	0,459525	-0,7774	-0,42389	0,759259	0,47691	-0,65088	-0,68075	-0,43404	0,690603	-0,73582	-0,64634	-0,76934
**trans-zeaxanthin**	0,592649107	0,781419	-0,03096	0,386217	0,219079	-0,12361	0,354516	0,423664	1	0,367135	-0,17931	-0,57611	-0,16742	-0,16409	0,064929	0,119782	0,3079	0,353556	0,374425	0,038137	-0,02596	0,537632	-0,07389	-0,02034	0,263824	0,625851	-0,24277	-0,51603	-0,54792	0,454672	-0,3585	0,048791	-0,14515
**7-cis lutein**	-0,076695475	0,127057	-0,53749	-0,26166	-0,61615	-0,35833	-0,38759	-0,24923	0,367135	1	-0,47916	0,297572	-0,63796	0,190637	-0,15687	-0,24475	-0,21151	-0,33264	-0,37588	-0,20718	0,440319	0,046958	0,273954	0,440205	-0,1555	0,212146	0,179732	0,097988	0,088746	-0,26591	0,452767	0,63308	0,609414
**β-cryptoxanthin**	0,251701939	0,033854	0,827279	0,480394	0,63621	0,60789	0,556345	0,473606	-0,17931	-0,47916	1	0,145569	0,787015	-0,21347	0,661717	0,693989	0,544166	0,689769	0,576222	0,694208	-0,29509	-0,01432	-0,40202	-0,29586	0,28785	-0,15067	-0,30134	0,075129	0,036983	0,534193	-0,38387	-0,34396	-0,48611
**13-cis β-carotene**	-0,434821461	-0,59121	-0,14402	-0,42451	-0,34065	-0,20147	-0,47436	-0,40577	-0,57611	0,297572	0,145569	1	0,127566	-0,04535	0,294725	0,158443	0,044312	-0,28713	-0,4751	0,294326	0,413662	-0,35614	0,193649	0,4089	-0,41172	-0,63048	0,395104	0,439888	0,47462	-0,40004	0,448985	0,418819	0,339677
**13'-cis β-carotene**	0,234685171	0,018013	0,723589	0,521109	0,809493	0,414455	0,429918	0,377299	-0,16742	-0,63796	0,787015	0,127566	1	-0,58517	0,723371	0,712759	0,747988	0,603938	0,454396	0,766699	-0,26283	0,186757	-0,20823	-0,26063	-0,06399	-0,23065	-0,16846	-0,04788	-0,05723	0,513365	-0,33396	-0,32035	-0,52403
**α-carotene**	0,040855359	0,05332	-0,22607	-0,33075	-0,59069	0,240615	-0,29625	-0,17845	-0,16409	0,190637	-0,21347	-0,04535	-0,58517	1	-0,27737	-0,25978	-0,69383	-0,27023	-0,29328	-0,31402	0,072409	-0,51586	-0,28339	0,065146	0,324476	-0,10922	-0,02702	-0,03297	-0,00783	-0,2738	-0,0078	-0,04786	0,038088
**trans β-carotene**	0,370286693	0,17589	0,628992	0,592082	0,5598	0,485847	0,474325	0,465895	0,064929	-0,15687	0,661717	0,294725	0,723371	-0,27737	1	0,959376	0,746775	0,757441	0,510567	0,997322	0,052107	0,302978	-0,31867	0,053676	0,182696	-0,09097	-0,25037	-0,18496	0,017391	0,523487	-0,34133	-0,09489	-0,52166
**9-cis β-carotene**	0,414757368	0,146083	0,674685	0,60882	0,652612	0,531906	0,595695	0,504424	0,119782	-0,24475	0,693989	0,158443	0,712759	-0,25978	0,959376	1	0,792988	0,827368	0,622475	0,965157	0,110712	0,359724	-0,27371	0,112855	0,270897	-0,06826	-0,3496	-0,19923	0,021451	0,545653	-0,40161	-0,12595	-0,55089
**9'-cis β-carotene**	0,344363846	0,16076	0,531272	0,494037	0,73157	0,15756	0,567147	0,409146	0,3079	-0,21151	0,544166	0,044312	0,747988	-0,69383	0,746775	0,792988	1	0,696411	0,574783	0,771632	-0,04851	0,458035	0,053971	-0,04306	-0,02883	0,096267	-0,20098	-0,19227	-0,16861	0,622165	-0,34845	-0,1151	-0,38218
**total carotenoids**	0,583806795	0,44717	0,823499	0,822068	0,746385	0,584353	0,913241	0,829394	0,353556	-0,33264	0,689769	-0,28713	0,603938	-0,27023	0,757441	0,827368	0,696411	1	0,937657	0,759853	-0,27779	0,497143	-0,50555	-0,27355	0,609809	0,384987	-0,58647	-0,4006	-0,19121	0,778857	-0,6495	-0,44672	-0,72915
**total lutein**	0,460062271	0,39185	0,743982	0,791152	0,705153	0,501195	0,995604	0,812188	0,374425	-0,37588	0,576222	-0,4751	0,454396	-0,29328	0,510567	0,622475	0,574783	0,937657	1	0,517488	-0,35826	0,519196	-0,42192	-0,35304	0,664024	0,560782	-0,63947	-0,36289	-0,16596	0,713676	-0,63694	-0,495	-0,66775
**total β-carotene**	0,359355353	0,145968	0,652022	0,588326	0,597993	0,487261	0,482894	0,461168	0,038137	-0,20718	0,694208	0,294326	0,766699	-0,31402	0,997322	0,965157	0,771632	0,759853	0,517488	1	0,041385	0,297324	-0,3021	0,043017	0,161782	-0,11828	-0,24904	-0,16648	0,022493	0,526669	-0,34326	-0,11031	-0,52648
**α-tocopherol**	-0,264361233	-0,42363	-0,42183	-0,21343	-0,21974	-0,15447	-0,35833	-0,42709	-0,02596	0,440319	-0,29509	0,413662	-0,26283	0,072409	0,052107	0,110712	-0,04851	-0,27779	-0,35826	0,041385	1	0,184845	0,4832	0,999939	-0,28872	-0,40694	0,152788	0,319641	0,536739	-0,5569	0,559778	0,807035	0,520877
**γ-tocopherol**	0,369525122	0,303104	0,337494	0,668265	0,608264	0,146954	0,487461	0,459525	0,537632	0,046958	-0,01432	-0,35614	0,186757	-0,51586	0,302978	0,359724	0,458035	0,497143	0,519196	0,297324	0,184845	1	-0,00213	0,194913	0,272872	0,352192	-0,1346	-0,14388	0,137581	0,470048	0,00412	0,097736	-0,08612
**δ-tocopherol**	-0,670245599	-0,55749	-0,60985	-0,35369	-0,20934	-0,35315	-0,37474	-0,7774	-0,07389	0,273954	-0,40202	0,193649	-0,20823	-0,28339	-0,31867	-0,27371	0,053971	-0,50555	-0,42192	-0,3021	0,4832	-0,00213	1	0,485787	-0,758	-0,12523	0,40113	0,596498	0,401502	-0,44332	0,678233	0,698617	0,755033
**total tocopherols**	-0,26234819	-0,42112	-0,41919	-0,20689	-0,21315	-0,15386	-0,35324	-0,42389	-0,02034	0,440205	-0,29586	0,4089	-0,26063	0,065146	0,053676	0,112855	-0,04306	-0,27355	-0,35304	0,043017	0,999939	0,194913	0,485787	1	-0,28807	-0,40193	0,152549	0,319495	0,537794	-0,55145	0,560583	0,807881	0,521232
**N**	0,559721798	0,421141	0,562107	0,392725	0,233039	0,363116	0,649548	0,759259	0,263824	-0,1555	0,28785	-0,41172	-0,06399	0,324476	0,182696	0,270897	-0,02883	0,609809	0,664024	0,161782	-0,28872	0,272872	-0,758	-0,28807	1	0,357335	-0,42439	-0,43214	-0,14964	0,494084	-0,59784	-0,56705	-0,59126
**P2O5**	0,256619546	0,585215	0,06247	0,468988	0,051876	0,070766	0,565638	0,47691	0,625851	0,212146	-0,15067	-0,63048	-0,23065	-0,10922	-0,09097	-0,06826	0,096267	0,384987	0,560782	-0,11828	-0,40694	0,352192	-0,12523	-0,40193	0,357335	1	-0,55456	-0,43003	-0,387	0,358982	-0,33347	-0,24343	-0,19797
**K2O**	-0,418971271	-0,39154	-0,39402	-0,51946	-0,25159	-0,42002	-0,62488	-0,65088	-0,24277	0,179732	-0,30134	0,395104	-0,16846	-0,02702	-0,25037	-0,3496	-0,20098	-0,58647	-0,63947	-0,24904	0,152788	-0,1346	0,40113	0,152549	-0,42439	-0,55456	1	0,562238	0,333384	-0,16047	0,517134	0,370953	0,525327
**Ca**	-0,755493745	-0,72365	-0,19373	-0,22523	-0,13642	0,091324	-0,33903	-0,68075	-0,51603	0,097988	0,075129	0,439888	-0,04788	-0,03297	-0,18496	-0,19923	-0,19227	-0,4006	-0,36289	-0,16648	0,319641	-0,14388	0,596498	0,319495	-0,43214	-0,43003	0,562238	1	0,823406	-0,32395	0,786449	0,583881	0,718036
**Mg**	-0,649821748	-0,75795	-0,05116	-0,03994	-0,07321	0,194088	-0,15234	-0,43404	-0,54792	0,088746	0,036983	0,47462	-0,05723	-0,00783	0,017391	0,021451	-0,16861	-0,19121	-0,16596	0,022493	0,536739	0,137581	0,401502	0,537794	-0,14964	-0,387	0,333384	0,823406	1	-0,36034	0,730886	0,541809	0,53937
**Fe**	0,6695895	0,599319	0,758095	0,651515	0,70823	0,434987	0,682021	0,690603	0,454672	-0,26591	0,534193	-0,40004	0,513365	-0,2738	0,523487	0,545653	0,622165	0,778857	0,713676	0,526669	-0,5569	0,470048	-0,44332	-0,55145	0,494084	0,358982	-0,16047	-0,32395	-0,36034	1	-0,55708	-0,53083	-0,56455
**Zn**	-0,646122704	-0,58454	-0,4838	-0,33038	-0,38844	-0,13703	-0,63635	-0,73582	-0,3585	0,452767	-0,38387	0,448985	-0,33396	-0,0078	-0,34133	-0,40161	-0,34845	-0,6495	-0,63694	-0,34326	0,559778	0,00412	0,678233	0,560583	-0,59784	-0,33347	0,517134	0,786449	0,730886	-0,55708	1	0,776443	0,925133
**Mn**	-0,497358822	-0,35928	-0,60167	-0,19696	-0,32969	-0,21332	-0,49282	-0,64634	0,048791	0,63308	-0,34396	0,418819	-0,32035	-0,04786	-0,09489	-0,12595	-0,1151	-0,44672	-0,495	-0,11031	0,807035	0,097736	0,698617	0,807881	-0,56705	-0,24343	0,370953	0,583881	0,541809	-0,53083	0,776443	1	0,800035
**Cu**	-0,612466673	-0,47641	-0,64674	-0,48166	-0,51796	-0,33524	-0,65277	-0,76934	-0,14515	0,609414	-0,48611	0,339677	-0,52403	0,038088	-0,52166	-0,55089	-0,38218	-0,72915	-0,66775	-0,52648	0,520877	-0,08612	0,755033	0,521232	-0,59126	-0,19797	0,525327	0,718036	0,53937	-0,56455	0,925133	0,800035	1
